# Rapid growth inhibitory activity of a YafQ-family endonuclease toxin of the *Helicobacter pylori tfs4* integrative and conjugative element

**DOI:** 10.1038/s41598-020-72063-x

**Published:** 2020-10-23

**Authors:** Kwadwo Boampong, Stephanie L. Smith, Robin M. Delahay

**Affiliations:** 1 Nottingham Digestive Diseases Centre, School of Medicine, University of Nottingham, Nottingham, NG7 2UH UK; 2 Present Address: Department of Theoretical and Applied Biology, Kwame Nkrumah University of Science and Technology, Kumasi, Ghana

**Keywords:** Biochemistry, Biological techniques, Microbiology, Molecular biology, Pathogenesis

## Abstract

Prokaryotic and archaeal chromosomes encode a diversity of toxin–antitoxin (TA) systems that contribute to a variety of stress-induced cellular processes in addition to stability and maintenance of mobile elements. Here, we find DinJ-YafQ family TA systems to be broadly distributed amongst diverse phyla, consistent with other ParE/RelE superfamily TAs, but more unusually occurring as a multiplicity of species-specific subtypes. In the gastric pathogen *Helicobacter pylori* we identify six distinct subtypes, of which three are predominantly associated with the mobilome, including the disease-associated integrative and conjugative element (ICE), *tfs4*. Whereas, the ICE-encoded proteins have characteristic features of DinJ-YafQ family Type II TA systems in general, the toxin component is distinguished by a broad metal-ion-dependent endonuclease activity with specificity for both RNA and DNA. We show that the remarkably rapid growth inhibitory activity of the ICE toxin is a correlate of a C-terminal lysine doublet which likely augments catalytic activity by increasing the positive electrostatic potential in the vicinity of the conserved active site. Our collective results reveal a structural feature of an ICE TA toxin that influences substrate catalysis and toxin function which may be relevant to specific TA-mediated responses in diverse genera of bacteria.

## Introduction

Toxin–antitoxin systems are virtually ubiquitous amongst bacteria and archaea and commonly encoded on plasmids and bacteriophages from which they can be disseminated, acquired and stably integrated into host chromosomes^1–4^. They were first revealed to play a role in plasmid maintenance^2^, however, chromosomally-encoded TA systems in particular are now recognised to have numerous important physiological roles which promote bacterial survival, including formation of a persister state, inhibition of bacteriophage, regulation of stress responses, biofilm formation and pathogenesis^5–11^.

Currently, seven distinct classes of TA system (Types I–VII) have been described based on the mechanism by which the antitoxin inhibits the activity of the toxin^12,13^. Whereas, the toxins associated with all classes tends to be proteins, the antitoxin is either also a protein (Types II, IV–VII) or a non-coding RNA (Types I and III). As a common mechanistic theme, the toxin of each TA pair causes growth arrest by interference with a critical cellular process in response to a particular environmental stress or vulnerability (e.g. viral predation) but is otherwise inhibited by antitoxin during normal growth. Liberation of toxin activity from antitoxin repression occurs as a consequence of the selective stress-induced degradation of the intrinsically more unstable antitoxin component^12^.

Type II TA systems, in which the toxin is inhibited by direct protein–protein interaction with the antitoxin, are particularly abundant and are represented by numerous conserved families including RelBE, CcdAB, YefM-YoeB, DinJ-YafQ, MazEF and ParDE^14–18^. Type II toxins tend to be endoribonucleases (RNases) which inhibit translation by related, but distinct mechanisms. These involve ribosome-dependent^18–20^ or independent cleavage of mRNA^21^ or the inhibition of ribosome-associated factors^22^, although exceptionally, ParDE family toxins inhibit replication by interference with DNA gyrase function^16^.

In the DinJ-YafQ system, the YafQ toxin is a ribosome-associating endoribonuclease in vivo which also exerts potent ribosome-independent cleavage activity in vitro^18^. Structural and mutagenesis studies have defined specific residues of YafQ important for toxicity, many of which are required for substrate recognition, binding and catalysis^19,23^. These include several active site histidine residues, His50, His63, His87 in addition to Asp67, Phe91 and a subset of positively charged, mainly lysine residues, which comprise extended basic surface patches^19^. The catalytic residues in particular are highly conserved between YafQ and two structurally homologous endoribonuclease TA toxins, HP0892 and HP0894 of *Helicobacter pylori*^24–27^. Four different Type II TA systems have been described so far in *H. pylori*, HP0892-HP0893, HP0894-HP0895, HP0315-HP0316 and HP0968-HP0967, the latter two comprising toxins of the virulence-associated protein family, VapD^28,29^.

*H. pylori* is a clinically important member of the Epsilon-proteobacteria which persistently colonises the gastric mucosa of ~ 50% of the World’s population. Although most colonised individuals remain asymptomatic, infection is a significant risk factor for several gastrointestinal diseases, including peptic ulcer disease and gastric cancer, the third leading cause of cancer-related deaths worldwide^30,31^. Disease susceptibility is considered to be multifactorial, involving a complex interplay between multiple environmental, host and bacterial factors. Of the latter, several well characterised virulence factors of *H. pylori* are known to contribute to the pathogenic potential of different *H. pylori* strains^32–34^ and more recently, components encoded on each of two variably present integrative and conjugative elements (ICEs) termed *tfs3* and *tfs4* have also emerged as potentially important in the host pathogen interaction^35–38^. ICEs are widespread amongst bacteria and commonly confer attributes such as resistance to antibiotics or new metabolic or virulence functions which increase fitness, genetic diversity and drive evolutionary change. ICEs also encode for conserved core activities which enable their chromosomal integration, excision and transfer to a recipient cell. When integrated, ICEs are stably maintained and replicated together with the host chromosome but are susceptible to loss if excision coincides with cell division^39^. However, similar to plasmids, ICEs also encode factors, including TA systems, which function to reduce the incidence of ICE-free daughter cells^39,40^.

In this study, we determine that DinJ-YafQ family TA systems are highly prevalent in prokaryotic genomes and unusually diversified into multiple species-specific subtypes. In *H. pylori*, we identify the variable presence of six distinct subtypes which includes HP0892-HP0893, HP0894-HP0895 in addition to three TAs encoded by either plasmids or the *tfs4* ICE. Focusing on the ICE TA system, we show that the associated toxin is structurally homologous to the chromosomal toxins HP0892 and HP0894, and although similar with respect to endoribonuclease activity, has a broader substrate range which includes both RNA and DNA. Moreover, the ICE toxin exerts a rapid and potent growth inhibitory activity relative to the other TA toxins which we determine is a correlate of a double lysine extension at the C-terminus of the protein. This appears to extend the surface area and positive electrostatic potential in the vicinity of the active site for recruitment of nucleic acid substrates, revealing a novel structure–function adaptation that efficiently modulates TA toxin activity.

## Results

### The *tfs4* ICE TA is a distinct subtype of the DinJ-YafQ-family

Up to five Type II TA systems have been identified in *H. pylori*^29^ of which two, HP0892–HP0893 and HP0894–HP0895 are members of the DinJ-YafQ family^24,25^. In a survey of *tfs* ICE content^38^, we identified a third putative DinJ-YafQ system suggesting that this family is particularly well-represented in *H. pylori* strains. Since the distribution and abundance of TA systems varies widely due to a tendency for both frequent horizontal gene transfer and intragenomic recombination^1, 4^ we were interested to assess the distribution of these modules relative to each other in the *H. pylori* population.

**Figure 1:**
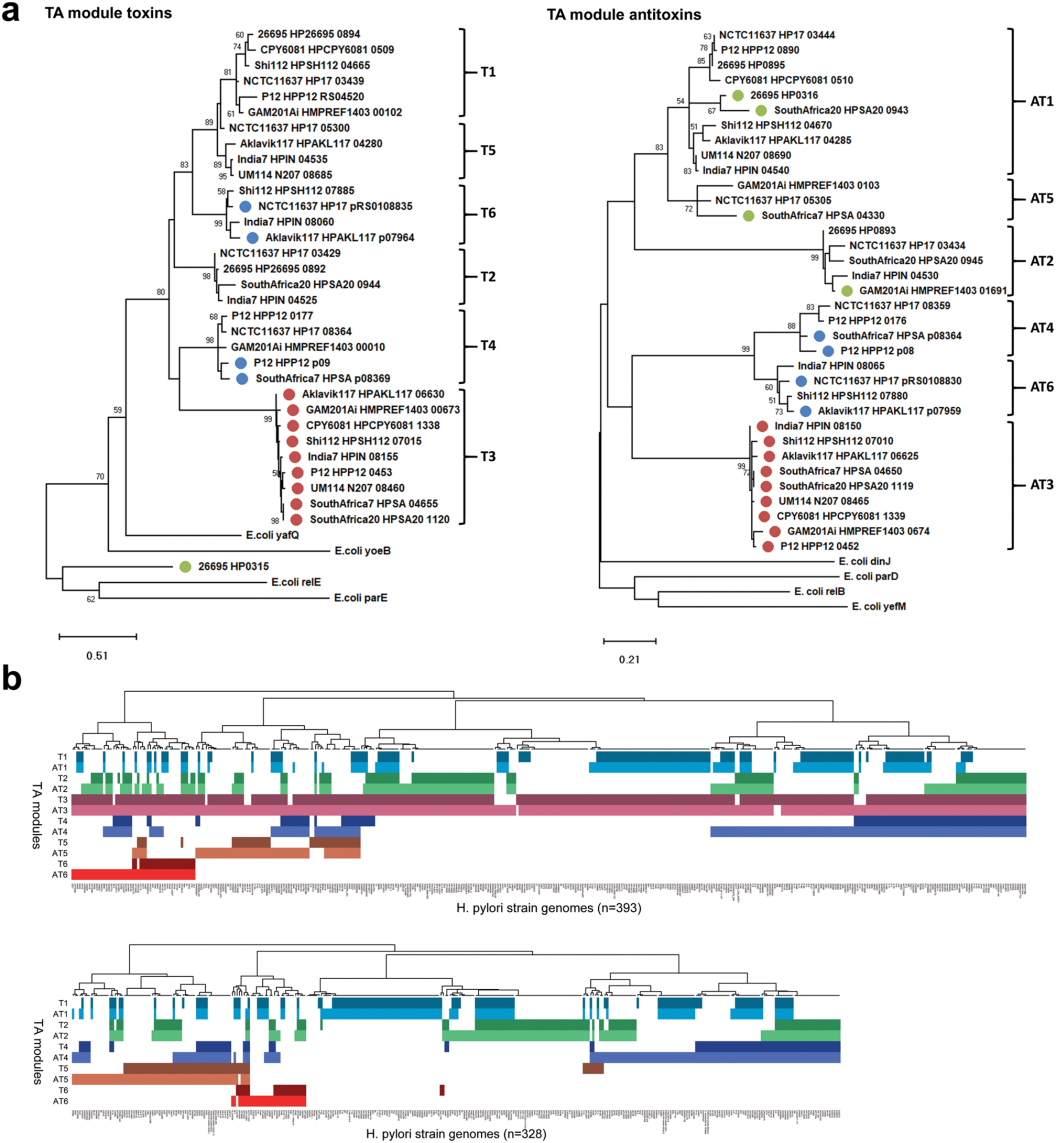
Phylogeny, prevalence and sequence conservation of *H. pylori* YafQ-family Type II toxin–antitoxin modules. (**a**) Phylogenetic analysis of *H. pylori* YafQ-family Type II TA module toxin and antitoxin nucleotide sequence variation using the Maximum Likelihood method. Toxin and antitoxin clades comprising representative TA subset sequences are labelled T1–T6 and AT1–AT6 respectively and include Type II antitoxin sequences associated with the non-Type II *vapD* toxin locus. TAs clearly associated with mobile elements or the *vapD* loci are indicated by coloured circles (blue; plasmid, red; *tfs4* ICE, green; *vapD* loci). The percentage (> 50%) of replicate trees in which the associated sequences clustered together in the bootstrap test (1,000 replicates) are shown next to the branches. (**b**) Prevalence of Type II TA modules in a global population of *H. pylori* strains. Hierarchical clustering indicates the distribution and co-occurrence of TA toxin subsets in both the presence (top, 393 strains) and absence (bottom, 328 strains) of the *tfs4* ICE encoded Clade T3/AT3 TA module.

For this, the putative ICE toxin sequence was initially used as query in a series of BLASTp searches to compile a dataset of 707 *H. pylori* consensus sequences from a total of 7,568 multispecies BLASTp subject hits. Surprisingly, subsequent phylogenetic analyses identified a total of six distinct clades (T1–T6) of *H. pylori* proteins nested within the YafQ-family of Type II TA toxins (Supplementary Fig. S1a, Fig. 1a *left*), and a similar multiplicity of related, but species-specific toxins from multiple different phyla indicating the YafQ family to be unusually diverse (Supplementary File S1). Associated antitoxin sequences produced a similar tree topology of major clades (AT1–6) nested within the DinJ antitoxin family (Supplementary Fig. S1a, Fig. 1a *right*). Antitoxin sequences associated with the *vapD* locus, encoding the Clustered Regularly Interspaced Short Palindromic Repeats (CRISPR) associated Cas2-like VapD protein and a Type II TA antitoxin^28^ were similarly identified within the DinJ family clustering with AT1, AT2 or AT5 Clades, whereas unrelated *vapD* sequences (represented by HP0315) formed an outgroup to the YafQ-like toxins as expected. The *vapD* locus is considered to be a hybrid or intermediate TA-like system, however, we found the *vapD* gene to be frequently flanked by a conserved partial sequence common to all six YafQ toxin clades, suggesting that the HP0315-homologous *vapD* gene is a likely foreign insertion into particular *yafQ* loci.

Two TA clades associated with *H. pylori* plasmids (Clades T4/AT4 and T6/AT6) were also apparent in the genomes of the phylogenetically-related *Helicobacter acinonychis* and *Helicobacter cetorum* strains but no other *Helicobacter* species, although surprisingly homologous sequences (> 94% nucleotide sequence identity) were also detected in the Bacteroidetes bacterium *Muricauda olearia* and a plasmid from four strains of the Firmicute *Staphylococcus pseudintermedius* (Supplementary Fig. S1b) suggesting recent cross-phyla exchange of these particular TA modules.

We next searched the PATRIC database to examine the relative distribution of each TA subtype within a geographically diverse collection of 721 genome sequenced *H. pylori* strains. Either or both ICE Clade T3/AT3 sequences were found in a total of 393 genomes and of these, the intact ICE TA module was present in 366 (93%), which was broadly comparable with the prevalence/integrity of Clade T1/AT1 and T2/AT2 TA sequences (326/86% and 324/79% respectively). Clade T4/AT4 TAs were also abundant in these genomes but more frequently inactivated by mutational attrition of the toxin gene (347/55%). This was similarly the case for the least abundant Clade T6/AT6 and T5/AT5 TA sequences (85/52% and 160/55% respectively) (Fig. 1b). We found no correlation between distribution and co-residence of the different TA modules in either the presence (Fig. 1b, top panel) or absence of the ICE TA loci (Fig. 1b bottom panel) suggesting that activity of neither plasmid nor genomic TA systems are likely to be restrictive of ICE acquisition.

### The *tfs4* ICE encodes a functional Type II TA module

**Figure 2:**
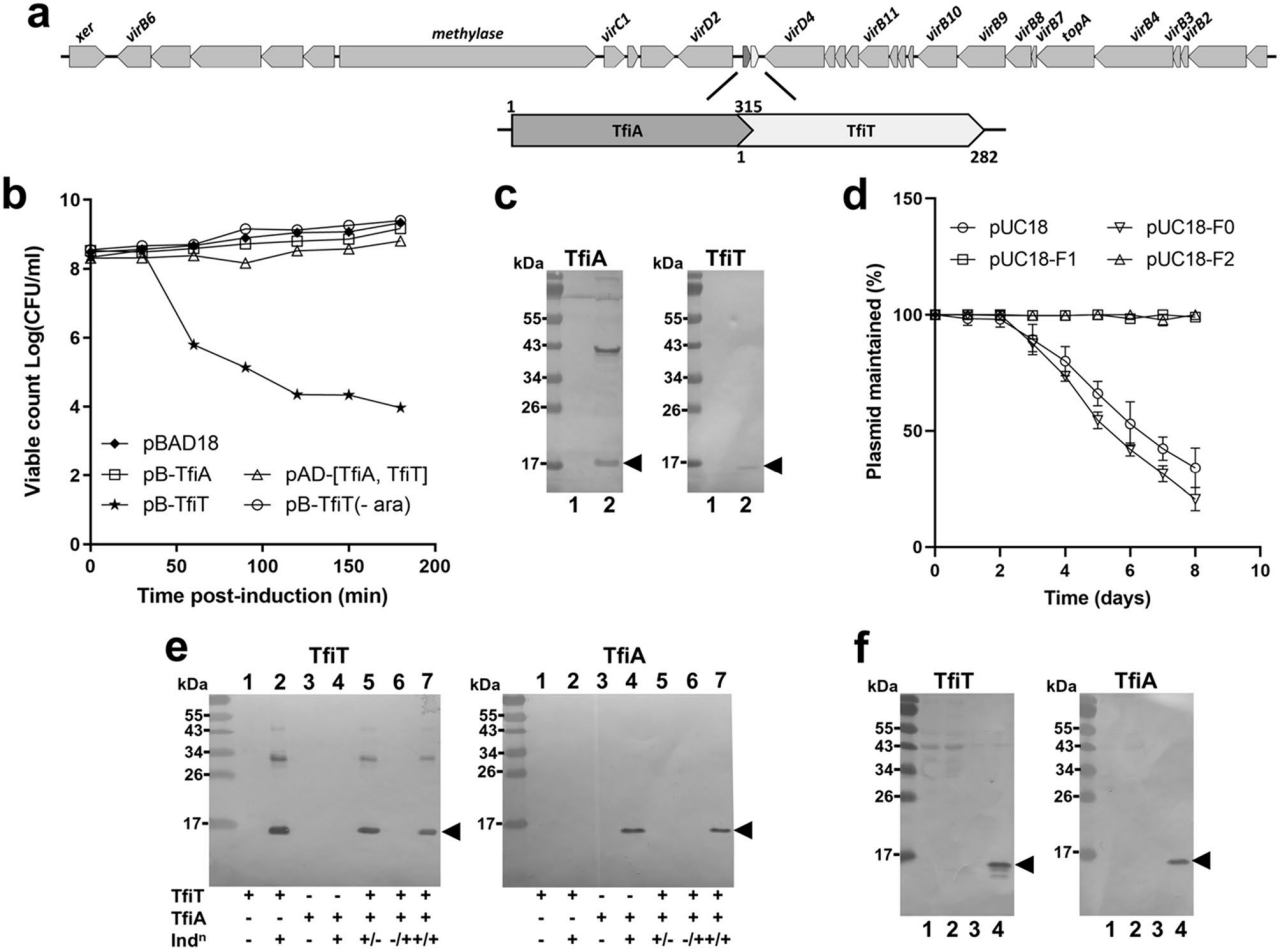
The *H. pylori tfs4* ICE encodes a functional Type II toxin–antitoxin system. (**a**) Conserved position of the putative TA module within the *tfs4* ICE gene cluster, comprising overlapping genes encoding toxin (denoted ‘TfiT’) and antitoxin (denoted ‘TfiA’). (**b**) Culture samples were taken at intervals post-induction for determination of viable counts. Expression of TfiT and TfiA individually was induced from pBAD18 constructs, whereas the TA module, comprising His-tagged TfiA and S-tagged TfiT was expressed from pACYCDuet-1 [TfiA, TfiT]. (-ara); no induction. (**c**) Relative expression of His-tagged TfiA or S-tagged TfiT (arrowheads) from the pACYCDuet-1 TA module construct in either uninduced (lanes 1) or induced (lanes 2) cultures. Western immunoblots were probed with either anti-His or anti-S-tag antibodies as appropriate. An unrelated ~ 40 kDa protein is detected in lysates by the His6 antibody. (**d**) Parent pUC18 or constructs comprising the entire TA module without (F0) or with either 213 bp (F1) or 160 bp (F2) of upstream sequence were passaged sequentially in the absence of antibiotic. Dilutions were made from daily cultures prior to passage for comparison of viable counts (CFU ml^−1^) on both selective and non-selective plates from which the percentage of plasmid maintained was calculated. (**e**) In parallel column-pulldowns, clarified lysate from induced (lanes 5, 7) or uninduced (lane 6) cultures of pBAD-TfiT were passed over TALON resin prior to addition of lysate from a culture of either induced (lanes 6, 7) or uninduced (lane 5) S-tagged-TfiA. Elution fractions were assessed for co-purified protein in Western immunblots probed with either anti-His-tag (left panel) or anti-S-tag antibody (right panel). Samples from uninduced/induced cultures for both TfiT (lanes 1, 2 respectively) and TfiA (lanes 3, 4 respectively) were included to monitor expression. (**f**) Far Western. Western blots containing resolved lysate from cultures of SoluBL21 (lane 1), SoluBL21/pACYCDuet (lane 2), and either uninduced (lane 3) or induced (lane 4) SoluBL21/pACYCDuet-TfiA (S-tagged TfiA) were incubated with purified His-tagged TfiT then either anti-His-tag (left panel) or anti-S-tag antibody (right panel). Arrowheads indicate TA proteins. Error bars in (**b**,**d**) display standard deviations from three replicates. *ara* arabinose, *Ind*^*n*^ induction.

In view of the evident conservation of the Clade T3/AT3 ICE modules within the *H. pylori* population we chose to investigate the *tfs4* TA locus further. Common to other Type II TA module proteins, both the ICE toxin and antitoxin proteins share physical properties of predicted overall charge and small size (MW 10.9 kDa/pI 9.1 and MW 12.2 kDa/pI 5.9 respectively), and are encoded by overlapping genes in an apparent bicistron located centrally within the *tfs4* ICE (Fig. 2a). In an initial investigation of the TA locus, we expressed the toxin, referred to here as ‘TfiT’ (*tfs*-four ICE toxin) for brevity, from the arabinose inducible pBAD18 plasmid using a selection of *Escherichia coli* strains as model hosts. In all cases, we observed a rapid post-induction reduction in growth along with a sharp decline in colony forming units (CFU ml^−1^) (Fig. 2b) consistent with TA toxin function. In contrast, no growth inhibitory activity was observed following similar expression of the putative antitoxin, ‘TfiA’ (*tfs*-four ICE antitoxin).

To examine if TfiA could inhibit TfiT activity, the entire encoding bicistron was engineered into the dual expression vector pACYCDuet-1 to enable both proteins to be epitope-tagged and co-expressed in the genetic context of the module. In this arrangement, TfiT toxicity was completely inhibited in the presence of TfiA (Fig. 2b), although it was notable that yields of the tagged proteins were not equivalent (Fig. 2c). We were unable to transform a control pACYCDUET-TfiT construct in these experiments, presumably due to leaky expression, therefore to confirm TfiA activity, we employed an alternative co-expression strategy in which delayed induction of TfiA (expressed from pACYCDuet-1 following induction on IPTG plates) was demonstrated to rescue cells from extended exposure to TfiT (expressed from pBAD18 in broth as before), whereas in the absence of antitoxin, TfiT-exposed cells were non-viable and could not be recovered (Supplementary Fig. S2). These results indicate that TfiT toxin activity is bacteriostatic and can be rescued in the presence of TfiA.

Since ICEs have both integrated and extrachromosomal states^39^, we next investigated the ability of the TA module to stabilise and maintain an extrachromosomal element. For this, we inserted the entire TA module without (F0) or with the inclusion of either a large (F1, 213 bp) or smaller (F2, 160 bp) stretch of upstream flanking sequence into the high copy number pUC18 plasmid. Sequential daily passage of plasmids without antibiotic selection in *E. coli* MG6155 showed complete retention of both F1/F2 TA-containing plasmids compared with a steady loss observed for the parent or F0 plasmids (Fig. 2d). These results demonstrate that functional activity of the ICE TA in vivo is sufficient to stably maintain an extrachromosomal element and moreover that TA expression is controlled by a native TA module promotor. In support of this latter observation, a promotor region comprising − 35 and − 10 boxes (sequences TTTTTT and GGCTATAAT respectively) is predicted to be within the first 65 bp of the antitoxin start codon in strain *H. pylori* P12.

Finally, as inhibition of toxin activity is mediated by direct interaction between toxin and antitoxin proteins in Type II TA systems^12,41^ we next assessed the ability of recombinant TfiT to capture and co-purify TfiA in a pulldown assay. For this, C-terminal His-tagged TfiT was immobilised on TALON metal affinity resin prior to incubation with lysate containing C-terminal S-tagged TfiA. Subsequent Western immunoblotting of test and control elution fractions showed that co-purification of TfiA was entirely dependent upon the presence of TfiA (Fig. 2e). In support of this observation, a Far Western approach using purified TfiT as a probe similarly showed specific binding to TfiA contained within a bacterial lysate (Fig. 2f). As final confirmation of the interaction, we used a series of yeast two-hybrid assays which showed a stable reciprocal pairwise interaction between TfiT and TfiA proteins and moreover, indicated a strong propensity for TfiA dimerization (Table 1).

**Table 1:** Yeast-two hybrid protein–protein interactions.

Bait^a^	TfiA		TfiT
Prey^b^	TfiA	TfiT	TfiA
−His/−Ade^c^	+++	+++	+++
−His^c^	+++	+++	+++
*lacZ*^d^ (Miller UNITS)	128.7 ± 8.6	60.8 ± 5.9	89.8 ± 7.4
*lacZ*^d^ (fold increase)^e^	27.4	12.9	47.2

^a^Bait and ^b^prey fusions were constructed in pGBT9 and pGAD424 vectors respectively.

^c^YMM plates were supplemented with Met and uracil and lacked either His, or both His and Ade as indicated.

^d^*lacZ* reporter activity was assessed by β-galactosidase assay.

^e^Fold increase relative to self-activation control for each bait vector.

Collectively, these results indicate that TfiA is the cognate antitoxin of TfiT and that TfiA–TfiT have features typical of other Type II TA systems.

### TfiT is a metal-ion-dependent endonuclease

**Figure 3:**
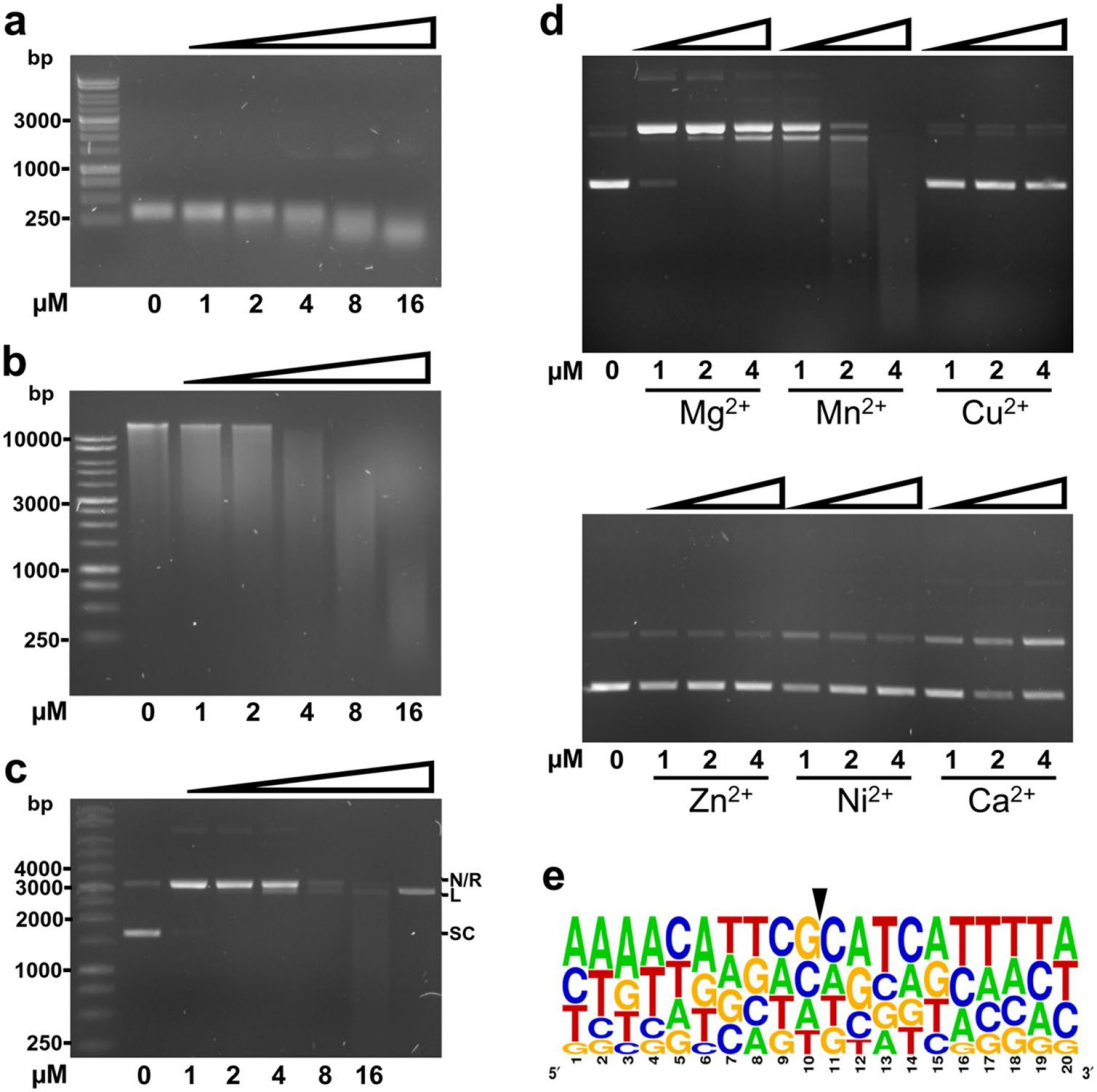
Broad endonuclease activity of TfiT**.** Incubation of purified TfiT-His toxin (1–16 µM) with (**a**) *H. pylori recA* mRNA (100 ng), (**b**) genomic DNA of *H. pylori* strain P12 (100 ng) and (**c**) pGEM-TEasy plasmid (100 ng) in reaction buffer (20 mM Tris–HCl pH 7.0, 50 mM NaCl, 2.5 mM MgSO_4_) at 37 °C for 30 min. A sample of *Nco*I-restricted linear plasmid DNA (30 ng) is included as a size marker in the final lane of (**c**). (**d**) Incubation of purified TfiT-His toxin (1–16 µM) with pGEM-TEasy plasmid (100 ng) in reaction buffer (20 mM Tris–HCl pH 7.0, 50 mM NaCl) supplemented with 2.5 mM of either MgCl_2_, MnCl_2_, CuCl_2_, ZnCl_2_, NiCl_2_ or CaCl_2_ at 37 °C for 30 min. (**e**) A selection of fragments generated by TfiT-cleavage (16 µM) of *H. pylori* strain P12 genomic DNA (250 ng) were sequenced, then mapped to the same strand of the P12 reference genome sequence to establish sequence context of the nick site. The WebLogo was generated from alignment of 42 informative sequences and shows the frequency of nucleotide bases up to 10 bp either side of the TfiT nick/cleavage site (indicated by an arrow). The relative height of each letter is indicative of the frequency of the indicated bases at each position. Labelled bands in (**c**) correspond to nicked/relaxed (N/R), linear (L) and supercoiled (SC) plasmid forms.

The *H. pylori* HP0892 and HP0894 Type II toxins are endoribonucleases which cleave mRNA with limited sequence specificity^24,25^. We therefore next sought to determine if TfiT similarly exhibited RNase activity by performing mRNA cleavage assays with purified His-tagged TfiT (0–16 uM). Results showed equivalent concentration-dependent degradation of two unrelated in vitro synthesised mRNA templates, *recA* (Fig. 3a) and *tfiT* (Supplementary Fig. S3a) consistent with endoribonuclease activity. To confirm the specificity of this activity towards RNA, we examined the ability of TfiT to degrade a variety of other nucleic acid substrates. However, contrary to expectation, TfiT demonstrated a similar concentration-dependent activity towards both genomic and plasmid DNA (Fig. 3b,c respectively) which was not apparent following pre-treatment of TfiT with Proteinase K (Supplementary Fig. S3b). With respect to plasmid, increasing concentration of TfiT showed a progressive conversion of supercoiled plasmid to relaxed and linear forms prior to more substantial degradation (Fig. 3c) consistent with single-stranded nicking activity. Notably, very low concentrations of TfiT (< 0.25 µM) were required for initial plasmid relaxation (Supplementary Fig. S3c). All observed nuclease activity of TfiT was dependent upon the presence of divalent cation (Mg^2+^), although Mg^2+^ could be effectively substituted by Mn^2+^, with a concomitant two to fourfold potentiation of nuclease activity. Other common cofactors of catalytic activity, Cu^2+^, Zn^2+^, Ni^2+^ and Ca^2+^ were found to be either suppressive or unable to promote TfiT nicking activity in vitro (Fig. 3d).

The uniform fragmentation observed for both genomic and plasmid DNA at higher concentrations of TfiT (Fig. 3b,c) suggests that TfiT activity also has low sequence specificity. To explore this, we developed a protocol to precisely determine the nick site of TfiT-cleaved DNA template. As the substrate with the greatest complexity of sequence, genomic DNA was treated with TfiT to produce ~ 100–3,000 kbp fragments, then on the assumption that coincident nicking on both strands would frequently generate blunt cleavage products, fragments were A-tailed, cloned and sequenced. Insert sequences from 30 transformant colonies were subsequently mapped to the reference genome sequence, from which we unambiguously determined the cleavage context at 42 unique insert ends (Supplementary File S2). Comparative analysis of nick site sequences however failed to identify any sequence consensus other than a slightly increased frequency of guanine and cytosine at the nick site (Fig. 3e), confirming our initial observations that TfiT nicking/cleavage activity has low or no sequence specificity.

### TfiT toxicity and catalysis are potentiated by C-terminal lysines

**Figure 4:**
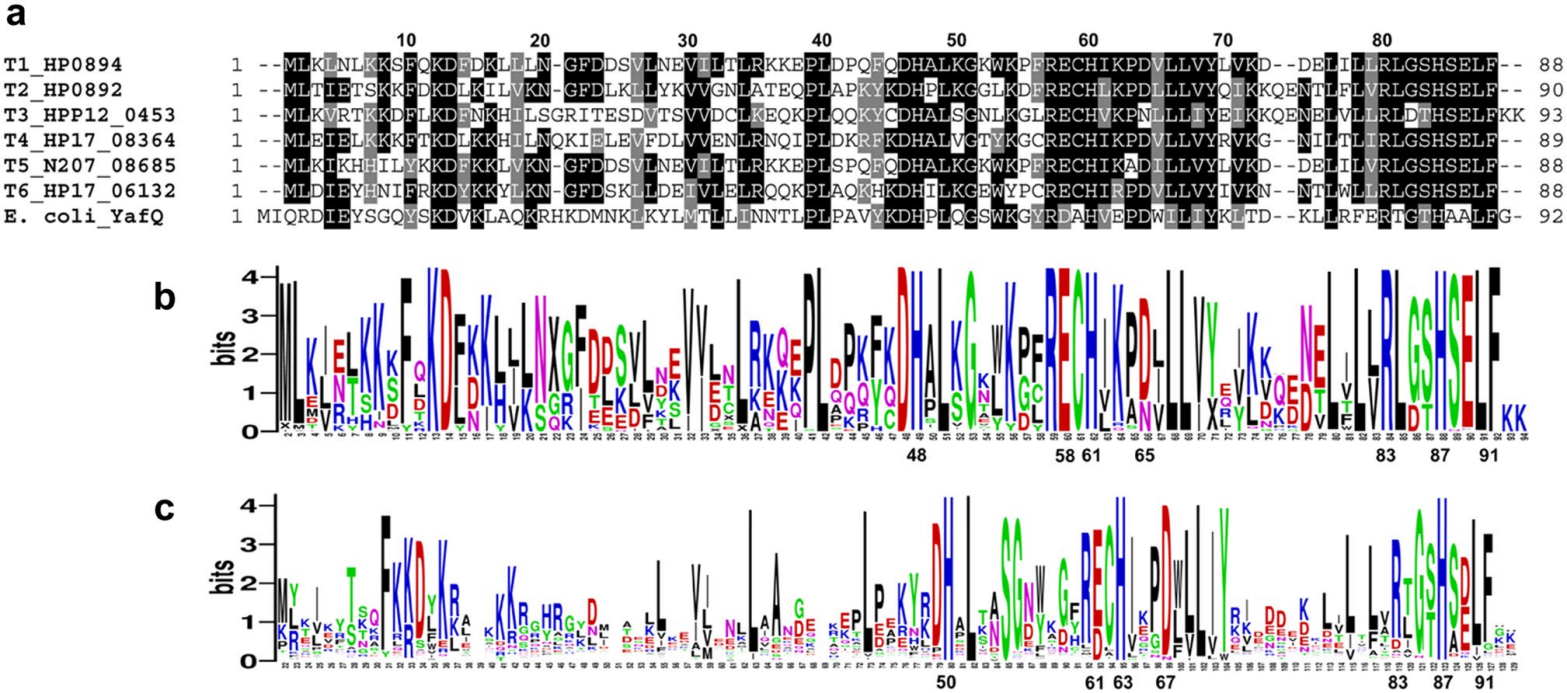
Sequence conservation of YafQ-family toxins. (**a**) Multiple sequence alignment of representative *H. pylori* Clade T1–T6 YafQ-family toxin sequences with *E. coli* YafQ. Numbering above the alignment refers to HP0894 sequence. WebLogos generated from multiple alignment of (**b**) Clade T1–T6 consensus sequences (n = 701) from a geographically diverse collection of *H. pylori* strains and (**c**) related Type II toxins from 318 other genera of bacteria. Sequences from multiple species from the majority of genera were included in the alignment to capture the full extent of toxin sequence diversity (n = 934 consensus sequences, Supplementary File S1). Numbering below each WebLogo refers to the position of conserved active-site residues His50, His63, Asp67, Arg83, His87 and Phe91 of *E. coli* YafQ (**c**) and the corresponding position of equivalent TfiT residues (**b**). In each logo, the height of individual amino acid letter codes corresponds to the prevalence of particular residues at each position in the protein sequence. A height of 4.2 bits is indicative of 100% conservation.

All six clades of *H. pylori* YafQ-family toxins show good conservation of sequence motifs including several active site residues within the C-terminal motif His/Ser/Glu/Leu/Phe (HSELF) (Fig. 4a,b) which are similarly well conserved in YafQ-family toxins from other diverse genera (Fig. 4c, Supplementary File S1). The His residue in particular (H87, H86, H84 of YafQ, HP0892 and HP0894 respectively) is a key catalytic residue, which together with the other HSELF residues comprise one of three clusters in the active site that are essential for recognition and binding of nucleotides^23,25,42^.

As the ‘HSELF’ motif is equally well conserved in TfiT, we constructed a series of C-terminal mutants including H87L and ΔHSELFKK (TfiT_Δ87–93_) to assess the functional homology of this motif. A further mutant, ΔKK (TfiT_Δ92–93_) was also included to investigate the contribution of the terminal lysine pair of TfiT, as this extension is distinctive of both the *H. pylori* Clade T3 ICE toxin and particular YafQ subtypes present in other genera (Fig. 4c, Supplementary File S1).

**Figure 5:**
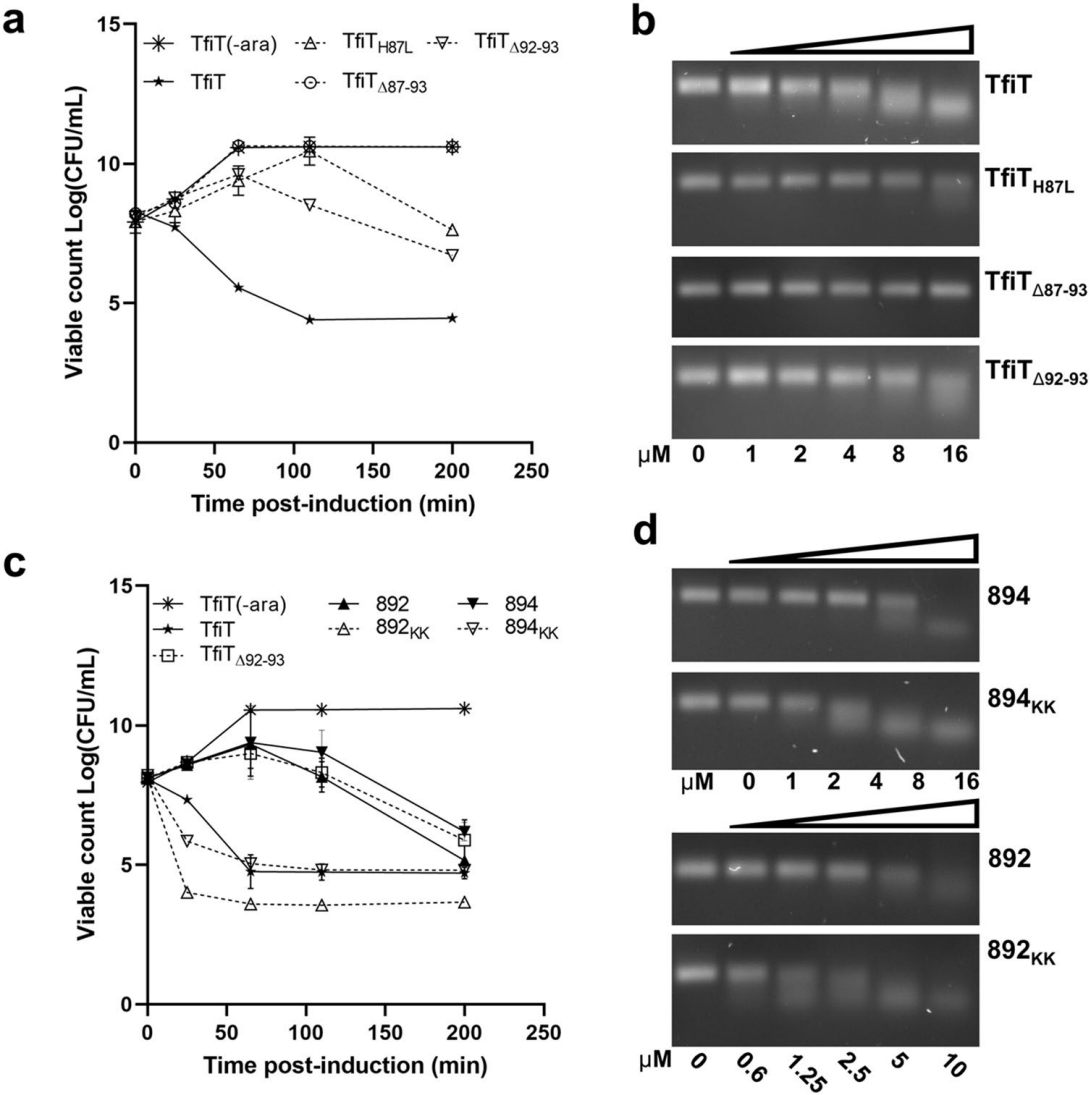
Effect of C-terminal mutation on the activity of TfiT, HP0892 and HP0894 toxins. (**a**) Samples from arabinose-induced cultures of pBAD18-TfiT wildtype and C-terminal mutants or (**c**) pBAD18-TfiT, pBAD18-HP0892, pBAD18-HP0894 and their respective derivatives either depleted of, or augmented with a C-terminal lysine doublet were taken at intervals for determination of viable counts. (**b**) Incubation of purified TfiT-His and C-terminal mutant derivatives (1–16 µM) or (**d**) TfiT-His, HP0892-His, HP0894-His and their respective C-terminal lysine-augmented or depleted derivatives (1–16 µM) with *H. pylori recA* mRNA (100 ng). Assays in (**b**,**d**) used standard reaction buffer (20 mM Tris–HCl pH 7.0, 50 mM NaCl, 2.5 mM MgSO_4_) and incubation conditions (37 °C for 30 min). Error bars in (**a**,**c**) display standard deviations from three replicates.

As observed for equivalent mutation of HP0894^25^, substitution of TfiT H87 markedly reduced, although did not entirely abolish RNase and growth inhibitory activity, whereas deletion of the entire TfiT C-terminal HSELFKK motif resulted in complete abrogation of all catalytic activity (Fig. 5a,b respectively). To verify the structural integrity of the TfiT_Δ87-93_ (HSELFKK) mutant protein, a column pulldown (Supplementary Fig. S4) and yeast two-hybrid assay (Supplementary Table S4) showed that C-terminal deletion did not compromise the stability or ability of recombinant TfiT_Δ87-93_ to interact with TfiA antitoxin.

Remarkably, deletion of the C-terminal lysine doublet in TfiT_Δ92–93_ was also seen to markedly decrease the toxicity of TfiT, reflected by a ~ 10^4^-fold initial increase in the viability (CFU ml^−1^) of TfiT_Δ92-93_ intoxicated cells, and also significantly delay its onset (Fig. 5a). Although less pronounced, a concomitant fold reduction in nuclease activity of the TfiT_Δ92–93_ mutant compared to wildtype TfiT was also apparent (Fig. 5b). These results therefore suggest that the presence of the C-terminal lysines enhances the activity of the wildtype ICE toxin.

To explore this further, we engineered an equivalent lysine doublet at the C-terminus of the Clade T2 and T1 genomic toxins HP0892 and HP0894, generating HP0892_KK_ and HP0894_KK_, and repeated growth experiments as before. Consistently, both wildtype HP0892 and HP0894 toxins, which naturally lack the lysine doublet (Fig. 4a), displayed a closely similar growth arrest phenotype as the TfiT_Δ92-93_ lysine mutant (Fig. 5c, Supplementary Fig. S5a), whereas addition of the lysine doublet to HP0894 increased its growth inhibitory activity to a level comparable with wildtype TfiT. The effect of the lysine doublet on HP0892 however was substantially more pronounced, incurring an almost immediate inhibition of growth and a > 10^3^-fold reduction in viability (CFU ml^−1^) of HP0892_KK_ intoxicated cells within the first 30 min of induction relative to TfiT (Fig. 5c, Supplementary Fig. S5a). Compared to the more modest increases observed for TfiT and HP0894_KK_, the HP0892_KK_ mutant also showed a > twofold potentiation of RNase activity relative to the wildtype toxin, confirming the overall enhanced effect (Fig. 5b,d, Supplementary Fig. S6).

Of additional note, whereas both HP0892 and HP0894 demonstrated some nicking of plasmid DNA, neither wildtype toxin, which were of similar purity and concentration as TfiT (Supplementary Fig. S5b) showed any discernible nuclease activity towards genomic DNA (Supplementary Fig. S5c). This was similarly the case for their respective lysine-augmented mutants, highlighting a fundamental difference in activity between the genomic and ICE-encoded toxins.

### Structural analysis of TfiT

**Figure 6:**
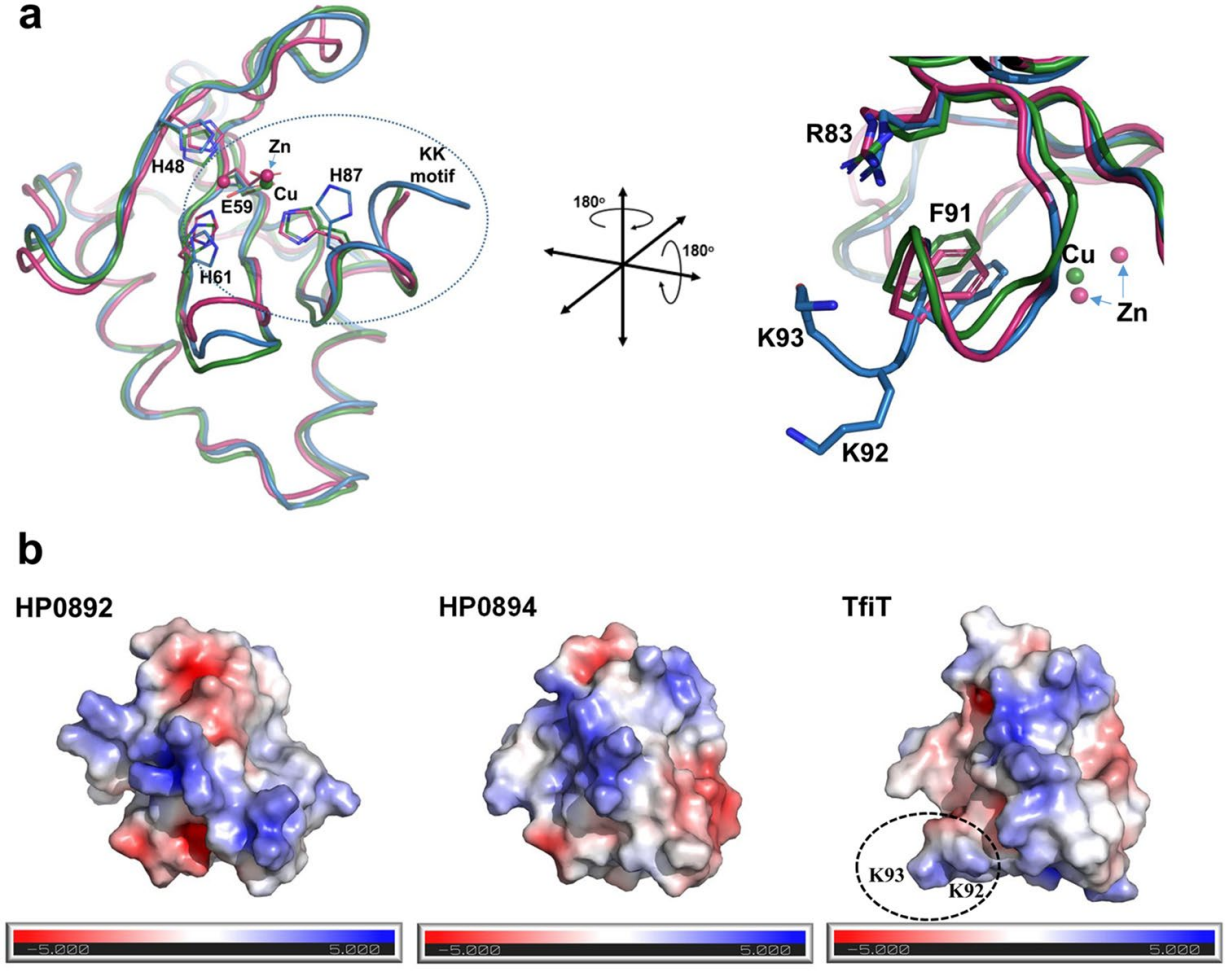
Structural homology of TfiT with genomic toxins HP0892 and HP0894. (**a**) Superposition of the crystal structures of Cu-bound HP0894 (PDB: 4LSY, green), Zn-bound-HP0892 (PDB: 4NRN, pink) and modelled TfiT (blue) highlighting side-chains of conserved active site residues important for acid–base catalysis during mRNA hydrolysis, shown in stick form, relative to the TfiT C-terminal lysine motif. (**b**) The C-terminal lysines of TfiT extend the electrostatic interface for substrate attraction and interaction. Surface representations of HP0894 (PDB: 4LSY), HP0892 (PDB: 4NRN) and modelled TfiT showing the electrostatic potential surface in the same orientation plane as (**a**), right. The accessible surface area is coloured according to calculated electrostatic potential from − 5 k_B_T/e (red) to + _B_T/e (blue).

To explore the impact of the C-terminal lysines on the structure of TifT we generated a homology model of TfiT using the structure of the related HP0894 toxin as template (PDB: 4LTT). Superposition of the TfiT homology model, initially with the structure of *H. pylori* HP0894 showed good correspondence and global maintenance of secondary structural elements (Supplementary Fig. S7), and together with HP0892 (PDB: 4NRN) confirmed the spatial conservation of key catalytic residues within the active site between all three molecules (Fig. 6a, left). In particular, the putative catalytic triad comprising E58/R82/H86 of HP0892 and E58/R80/H84 of HP0894^24^ is equally well-conserved in TfiT (E59/R83/H87) (Fig. 6a). The conserved Glu residue of the triad acts as a general base with His as a general acid in the acid–base catalysis reaction whereas Arg is involved in phosphate binding and stabilisation of the transition state^26,27^. In addition to these critical catalytic residues, aromatic side-chains of highly conserved Phe/Tyr residues in the extreme C-terminus of the toxins, including Phe88 in HP0894, Phe90 of HP0892, Phe91 of YafQ, and Tyr87 of RelE are involved in substrate orientation and facilitate the acid–base catalysis reaction^23,26,27,43^. The sequence and structural conservation of equivalent TfiT residues, including Phe91 (Fig. 6a right) suggests they are also likely to contribute to catalysis in these respects.

In the immediate context of these catalytic residues, the lysine doublet of TfiT appears to form an extension of the α3-helix relative to the C-terminus of HP0892 and HP0894, where their disposition on the helical turn locates them on the face of the molecule adjacent to the conserved Arg83 (Fig. 6a, right). In an electrostatic surface representation of the modelled TfiT structure it is apparent that whereas the presence of the lysines generally extends the surface area in the immediate vicinity of the active site, their basic side-chains also contribute to an increase in the local positive electrostatic environment (Fig. 6b). This might therefore provide a larger surface and increased attractive force for substrate interaction similar to the RelE model of catalysis, whereby electrostatic forces from basic side chains are proposed to draw mRNA substrate into the active site binding pocket^43^.

## Discussion

In this report, we have found Type II toxin–antitoxin systems of the DinJ-YafQ family to be widespread amongst diverse genera of bacteria and frequently occurring in a multiplicity of strain and species-specific subtypes. In *H. pylori*, six distinct subtypes can be identified, four of which are novel and not identified in a previous survey of Type II TA loci^29^. All six TA loci have the capacity for host chromosome integration, although two, represented by Clades T4/AT4 and T6/AT6, are commonly found on *H. pylori* plasmids and can presumably readily mobilise between strains. Interestingly, homologous T4/AT4 module sequences are also apparent on small WGS contigs of four *S. pseudintermedius* strains (Supplementary Fig. S1B) which also include sequences of two other hypothetical proteins similarly common to *H. pylori* plasmids. The high level of identity between these common sequences suggests a recent and hitherto unappreciated inter-species transfer of the encoding plasmids although the dynamics of such an interaction remains unclear since neither *H. pylori* nor *S. pseudintermedius*, a common skin inhabitant of domestic cats and dogs^44^, are known to reciprocally colonise the other’s host.

Surprising, the plasmid-associated TAs appeared inactive in nearly half of all strains in which they occurred due to the absence or mutation of the toxin gene. Possibly, this reflected a toxin-independent function of antitoxin as previously suggested for DinJ^45^, or some functional redundancy as a consequence of host integration of the TA module rather than a dispensable plasmid function. However, this could not be readily determined due to the often limited sequence context available from short encoding WGS contigs. In contrast, we found the TA module encoded by the *tfs4* ICE to be invariably intact and ancestrally conserved, arguing for an ongoing requirement specifically for the Clade T3/AT3 TA module in *tfs4* ICE function. However, that the TA module is often absent in remnant *tfs4* ICEs^38,46^ suggests that the TA system does not prevent ICE decay during long periods of stable integration. Indeed, acquisition/exchange, loss and degeneration of functional modules is a feature of ICE evolution^47^ which is similarly observed for *tfs4* in many *H. pylori* strains^38,46^. Our collective results determine that the characteristics and broad activity of TfiA and TfiT are consistent with features of the prototypical *E. coli* DinJ-YafQ TA system and both the HP0892/HP0893 and HP0894/HP0895 chromosomal TA pairs of *H. pylori*^18,24,25^. Most notably, (i) both genes overlap in a bicistron and characteristically encode acidic (TfiA) and basic (TfiT) small proteins which (ii) are phylogenetically related to the DinJ-YafQ family of Type II TA systems, (iii) TfiT strongly inhibits bacterial growth, (iv) TfiT-mediated growth arrest can be both inhibited and rescued in the presence of TfiA, (v) TfiA and TfiT specifically and stably interact together and (vi) TfiT has cation-dependent ribonuclease activity which (vii) requires a highly conserved C-terminal active site His residue, typical of YafQ-family toxins, for catalysis. In addition, the ability of TfiA-TfiT to maintain a constitutively extrachromosomal element (i.e. plasmid) from host loss in vivo further suggests that the ICE TA may confer an important stability function in respect of *tfs4* consistent with observations for other ICE TAs^40,48^ and *tfs4* itself^49^. However, although TfiA-TfiT are conventional in these respects several aspects of TfiT activity are distinctive. In particular, in addition to RNase activity, we also found the substrate range of TfiT to unexpectedly extend to DNA.

In a plasmid model, our cleavage assays clearly delineated a progressive nicking relaxation, linearization and comprehensive fragmentation of supercoiled plasmid suggesting that TfiT possesses both nicking endonuclease and RNase activities. Endonuclease activity was uniquely also demonstrated towards genomic DNA and both RNase/DNase activity could be abrogated by mutation or deletion of key C-terminal catalytic residues comprising the ‘HSELF’ motif. As these residues contribute to both generation of the active site binding pocket and catalysis in other YafQ-like toxins and the modelled TfiT, it can be considered that binding and catalysis of both RNA and DNA substrates occurs at the same or spatially close sites, consistent with other sugar non-specific nucleases^50,51^. Non-typical Type II toxins with DNA nicking endonuclease activity have been reported amongst the RelE/ParE toxin superfamily, most notably Vp1843 of *Vibrio parahaemolyticus*^52^. Similarly, the RalR toxin of the RalR/RalA Type I TA system functions as a non-specific endonuclease similar to DNase I^53^. Neither protein however shares sequence features or the substrate range of TfiT, although as nuclease toxins, both also appear to be rapidly inhibitory of bacterial cell growth.

Of particular note in this study, we determined that the C-terminal Lys91/Lys92 doublet peculiar to TfiT conferred a remarkable potentiation of its activity. The particular disposition of the lysine side-chains immediately proximal to the active site presents a highly basic surface which is likely to increase recruitment, presentation and subsequent catalysis of nucleic acid substrates. Neubauer et al*.* proposed a similar role for basic side-chains in the vicinity of the active site in a model of RelE catalysis^43^. It has also been established that lysine repeats in the C-terminus of enzymes such as topoisomerase I (TopA) are essential for DNA binding and enable high enzyme processivity through stabilisation of the DNA-enzyme complex^54^. The marked increase in RNase activity we observed with the lysine-augmented HP0892 toxin in particular suggests this may similarly be the case for TfiT and would account for its rapid and potent toxicity. In the context of the *tfs4* ICE, this might be required to provide a rapid safeguard against ICE loss following transient periods of ICE excision.

However, other determinants of substrate specificity presumably direct subsequent substrate recognition since neither HP0892_KK_ nor HP0894_KK_ toxins were also found to be catalytically active towards genomic DNA in our in vitro assays. In this respect, extended basic patches on the surface of *E. coli* YafQ and HP0894, comprising lysine residues Lys50, Lys52 and Lys54 are recognised to be particularly important for substrate interactions^19,25,26^, although are not especially well-conserved between other YafQ-family members, including TfiT (comprising Ser51, Asn53, Lys55 in equivalent positions) (Fig. 4). Therefore, the particular distribution of lysines is likely to introduce variability in the electrostatic surface of different toxins, as indicated in the electrostatic surface representation of the *H. pylori* YafQ toxins (Fig. 6b), which directly impacts binding and specific presentation of substrate at the active site.

Whilst TfiT is the only *H. pylori* YafQ-family subtype with a C-terminal lysine extension it is noteworthy that single or double lysines, in addition to a range of other residues with different charge and side-chain properties are also a common terminal feature of YafQ toxins in many other bacteria (Fig. 4c, Supplementary File S1). The findings of the present study suggest that these could similarly impact substrate interactions at the active site and may provide a common mechanism for precise functional modulation of toxin activity relative to different physiological roles of particular species-adapted YafQ-family TA systems.

## Methods

### Microbial strains and culture conditions

Strains used are listed in Supplementary Table S1. *H. pylori* strains were cultured with minimal passage on 5% (v/v) horse blood agar plates (Oxoid) at 37 °C in a microaerophilic atmosphere. *E. coli* strains were grown at 37 °C in Luria Broth (LB) or agar (Oxoid), supplemented with ampicillin (50–100 µg ml^−1^), kanamycin (50 µg ml^−1^), chloramphenicol (35 µg ml^−1^), l-arabinose (0.02% w/v) or isopropyl-β-d-thiogalactopyranoside (IPTG, 1 mM) as required. *Saccharomyces cerevisiae* was maintained in SC medium supplemented with 2% glucose (w/v) at 30 °C.

### Generation of recombinant plasmid constructs

Plasmids and constructs are listed in Supplementary Table S2. Genomic DNA was prepared from *H. pylori* strains using the GenElute Bacterial Genomic DNA kit (Sigma-Aldrich). Toxin, antitoxin and TA module sequences were PCR-amplified using Phusion DNA polymerase (New England BioLabs) using oligonucleotide primers (Sigma-Aldrich) listed in Supplementary Table S3. PCR products were restricted with NcoI/EcoRI, SacI/SalI or EcoRI/SalI for subsequent ligation into corresponding sites of the pBAD/*myc*-His, pUC18 or pGAD424/pGBT9 yeast-two hybrid vectors respectively. *tfiA* was ligated into NdeI/XhoI sites of the MCS2 of pACYCDuet-1 for fusion to the S-tag sequence (encoding the epitope KETAAAKFERQHMDS) and the entire TA module inserted across both MCS1/MCS2 of pACYCDuet-1 using BamHI/XhoI enzymes, enabling concomitant fusion of *tfiT* and *tfiA* with His6-tag and S-tag sequences respectively. Constructs were transformed to BL21(DE3) pLysS or SoluBL21 for overexpression of recombinant protein or additionally, MG1655 and BW25113 strains for assessment of phenotypic effects. All constructs were confirmed by DNA sequencing (Source BioScience).

### Toxicity and recovery assay

Cultures containing recombinant pBAD/*myc*-His plasmids were induced with 0.02% (w/v) l-arabinose at an optical density (OD_600_) of ~ 0.3. Samples taken at time-points were serially diluted then plated in triplicate on LB agar plates (± 100 µg ml^−1^ ampicillin) for calculation of colony forming units (CFU ml^−1^). Experiments involving pACYC-Duet-1 constructs in either BL21(DE3) pLysS or SoluBL21 were performed in the same way following IPTG (1 mM) induction. For recovery assays, cultures of SoluBL21 co-transformed with both pBAD-*tfiT* and pACYCDuet-*tfiA* constructs were induced with 0.02% (w/v) l-arabinose for 3 h, then diluted samples plated in triplicate on selective LB agar with or without IPTG (1 mM) prior to calculation of CFU ml^−1^ after 16 h incubation.

### Plasmid stability assay

A single colony of *E. coli* MG1655 containing pUC18, pUC18-F0, pUC18-F1 or pUC18-F2 was inoculated into 10 ml of LB broth without antibiotic then cultures passaged 1:2,000 every 24 h for 8–10 passages. At each passage, a 1,000,000 × dilution of overnight culture was also prepared and 75 µl spread onto LB agar plates without antibiotic. Plates were incubated at 37 °C overnight then replica-plated to fresh LB agar plates (± 100 µg ml^−1^ ampicillin). Following overnight incubation, the number of colonies on the selective plates were expressed as a percentage of total colonies on the non-selective plates as a measure of plasmid maintenance.

### Purification of recombinant toxins

Cultures of SoluBL21 containing pBAD/*myc*-His toxin constructs were grown to an OD_600_ of 0.6–0.7 prior to addition of 0.02% (w/v) l-arabinose for 3 h. Harvested cells were lysed by sonication in binding buffer containing 20 mM Tris–HCl pH 7.0, 200 mM NaCl, 5 mM imidazole and 1X protease inhibitors (Roche) using a Soniprep 150 sonicator (Sanyo). Lysates were centrifuged at 10,000×*g*, 0.45 µm-filtered, then incubated with TALON metal affinity resin (Clontech) for 1 h at 4 °C, prior to loading in gravity-flow columns. His-tagged proteins were eluted using binding buffer containing 300 mM imidazole. Elution fractions were immediately concentrated and buffer exchanged into storage buffer (25 mM Tris–HCl pH 7.5) using Vivaspin centrifugal concentrators (Sartorius Ltd) and stored at − 80 °C. Protein purity was estimated to be > 95% for all purified proteins by SDS-PAGE and protein concentrations determined using the BCA assay (Pierce).

### Pull-down assay and far western

Clarified lysate containing His-tagged protein was immobilised on TALON metal affinity resin (Clontech) prior to addition of lysate containing S-tagged protein. Following a comprehensive wash step, immobilised proteins were eluted in binding buffer containing 300 mM imidazole then samples resolved by 15% SDS-PAGE prior to Western blotting. Blocked blots were probed with either anti-His_6_ antibody (Novagen) or anti-S-tag antibody (ThermoFisher Scientific), then alkaline phosphatase-conjugated secondary antibody (Sigma-Aldrich) prior to signal detection using 5-bromo-4-chloro-3indolyl phosphate/nitro blue tetrazolium liquid substrate (Sigma-Aldrich). For Far Western assays, lysates containing S-tagged antitoxin were resolved by SDS-PAGE prior to transfer to nitrocellulose by Western blotting. Blocked blots were probed with purified His-tagged toxin protein (0.12 µg ml^−1^) overnight at 4 °C, washed, then probed with either anti-His_6_ antibody (Novagen) or anti-S-tag antibody and developed for signal detection as before.

### Yeast two-hybrid assay

Relevant pGBT9 and pGAD424 constructs (20 µl each) were co-transformed into *S. cerevisiae* strain PJ69-4A using the high efficiency lithium acetate transformation procedure^55^. Co-transformants were initially selected by plating on yeast minimal medium (YMM) supplemented with 2% glucose (w/v) plus methionine (20 µg ml^−1^), uracil (20 µg ml^−1^), histidine (20 µg ml^−1^), and adenine (20 µg ml^−1^) (MUHA plates) and then subsequently replica-plated onto YMM lacking either histidine (MUA) or both histidine and adenine (MU plates) to select for activation of the HIS3/ADE2 reporters. Quantitative assessment of β-galactosidase activity in cell extracts of PJ69-4A cell was made using *o*-nitrophenyl-β-d galactopyranoside as substrate^56^.

### Nucleic acids and cleavage assays

Dilutions of purified toxin (0–16 µM) were incubated with mRNA (100 ng), pGEM-TEasy plasmid (50 ng) and genomic DNA from *H. pylori* strain P12 (100 ng) in reaction buffer (20 mM Tris–HCl pH 7.0, 50 mM NaCl, 2.5 mM MgSO_4_) for 30 min at 37 °C. Reactions were quenched by addition of 6 × purple loading dye (New England BioLabs) then analysed by electrophoresis on 0.8% agarose gels stained with ethidium bromide. Purified NcoI-restricted linear plasmid DNA (30 ng) was optionally included as a size marker. Reaction buffer was also prepared with 2.5 mM of each of MgCl_2_, MnCl_2_, CuCl_2_, ZnCl_2_, NiCl_2_ and CaCl_2_ as required.

Relevant templates were prepared using the GenElute Bacterial Genomic DNA kit (Sigma-Aldrich), Nucleospin Plasmid DNA purification kit (Macherey–Nagel) or RNeasy Plus Mini kit (Qiagen) as appropriate. Messenger RNA was transcribed in vitro from PCR-amplified *recA* or *tfiT* template using the HiScribe T7 Quick High Yield RNA Synthesis kit (New England BioLabs). The quality and concentration of DNA and RNA samples was monitored with a Nanodrop spectrophotometer (Thermo Scientific).

### Nick-site determination

Purified genomic DNA (250 ng) from *H. pylori* strain P12 was incubated with TfiT toxin (16 µM) in reaction buffer (20 mM Tris–HCl pH 7.0, 50 mM NaCl, 2.5 mM MgSO_4_) for 30 min at 37 °C. Cleaved products were purified, then A-tailed by incubation with 0.2 mM dATP and 5U Taq DNA polymerase (New England BioLabs) at 70 °C for 20 min. The modified products were ligated with pGEM-TEasy then transformed into *E. coli* XLI-Blue. Plasmid DNA was isolated from 30 transformant colonies, inserts sequenced (Source BioScience), then sequences searched against the P12 genome sequence to determine orientation, flanking sequence context and precise cleavage site at both insert ends. The sequence 10 bp either side of the cleavage site was determined for each insert end, then all 20mers aligned using Clustal Omega. WebLogo^57^ was used to determine positional sequence conservation of aligned sequence subsets.

### Generation and analysis of datasets

Toxin and antitoxin sequences were retrieved from the NCBI non-redundant sequence database following BLASTp searches using default parameters (https://blast.ncbi.nlm.nih.gov/Blast.cgi). Subject sequences were depleted of major truncates using the BioEdit Sequence Editor^58^, then multiple sequence alignments performed using Clustal Omega (https://www.ebi.ac.uk/Tools/msa/clustalo/) for derivation of initial toxin and antitoxin sequence subsets. Promotor sites were predicted using the Softberry BPROM service (https://www.softberry.com/berry.phtml?topic=bprom&group=programs&subgroup=gfindb).

For TA module prevalence, representative TA sequences were used in sequential BLASTp searches of the *Helicobacteraceae* database within the PATRIC bioinformatics platform^59^. Subject hits were assigned to TA module subsets based on percentage identity cutoff (> 85% identity) and confidence scores, then sorted by PATRIC genome identifier to determine TA module representation within individual strain genomes. WebLogo^57^ was used to determine positional sequence conservation of the final aligned sequence subsets. Gene content matrices representing the prevalence of YafQ-family toxin–antitoxin genes in *H. pylori* genomes were built in R(v3.2.2)^60^ using the ‘gplots’ package^61^. Hierarchical clustering of loci used the ‘hclust’ function (ward.D2 method and Euclidean distance measure) to generate a sidelong dendrogram.

### Phylogenetic analyses

Sequences aligned with ClustalW or MUSCLE were used for generation of phylogenetic trees in MEGA X^62^. The evolutionary history was inferred using the Maximum Likelihood method and Tamura 3-parameter model with 1,000 bootstrap replicates. A discrete Gamma distribution was used to model evolutionary rate differences among sites.

### Homology modelling

Homology modelling of HPP12_0453 (TfiT) used the PyMod 2.0 plugin module for PyMol^63,64^ as a convenient interface to Modeller 9v4^65^. The *H. pylori* HP0894 structure (PDB: 4LTT) was used for primary alignment as the highest scoring template. Output models with corresponding low scoring Discrete Optimised Protein Energy (DOPE) profiles were manually inspected for agreement with secondary structural elements then assessed using PROCHECK^66^ and ERRAT^67^ prior to final model selection. Surface electrostatics were calculated using APBS and pdb2pqr from within PyMol^68,69^.

## Supplementary information

Supplementary Information 1.

Supplementary Information 2.

Supplementary Information 3.

## Data Availability

Additional datasets generated during the current study are available from the corresponding author.
