# Supplementary material for: Rapid growth inhibitory activity of a YafQ-family endonuclease toxin of the Helicobacter pylori tfs4 integrative and conjugative element
Source: Sci Rep. 2020 Oct 23;10:18171. doi: 10.1038/s41598-020-72063-x (PMC7584586; doi:10.1038/s41598-020-72063-x)
Supplement: Supplementary file 3 — Supplementary Information 3. [file 41598_2020_72063_MOESM3_ESM.docx]

**Supplementary Information**

**Rapid growth inhibitory activity of a YafQ-family endonuclease toxin of the *Helicobacter pylori tfs4* integrative and conjugative element.**

Kwadwo Boampong^1,2^, Stephanie L. Smith^1^ and Robin M. Delahay^1^

^1^Nottingham Digestive Diseases Centre, School of Medicine, University of Nottingham, Nottingham, NG7 2UH, UK. ^2^Present address: Department of Theoretical and Applied Biology, Kwame Nkrumah University of Science and Technology, Kumasi, Ghana.

Address correspondence to R.M.D. (email: [rob.delahayuon@gmail.com](mailto:rob.delahayuon@gmail.com))


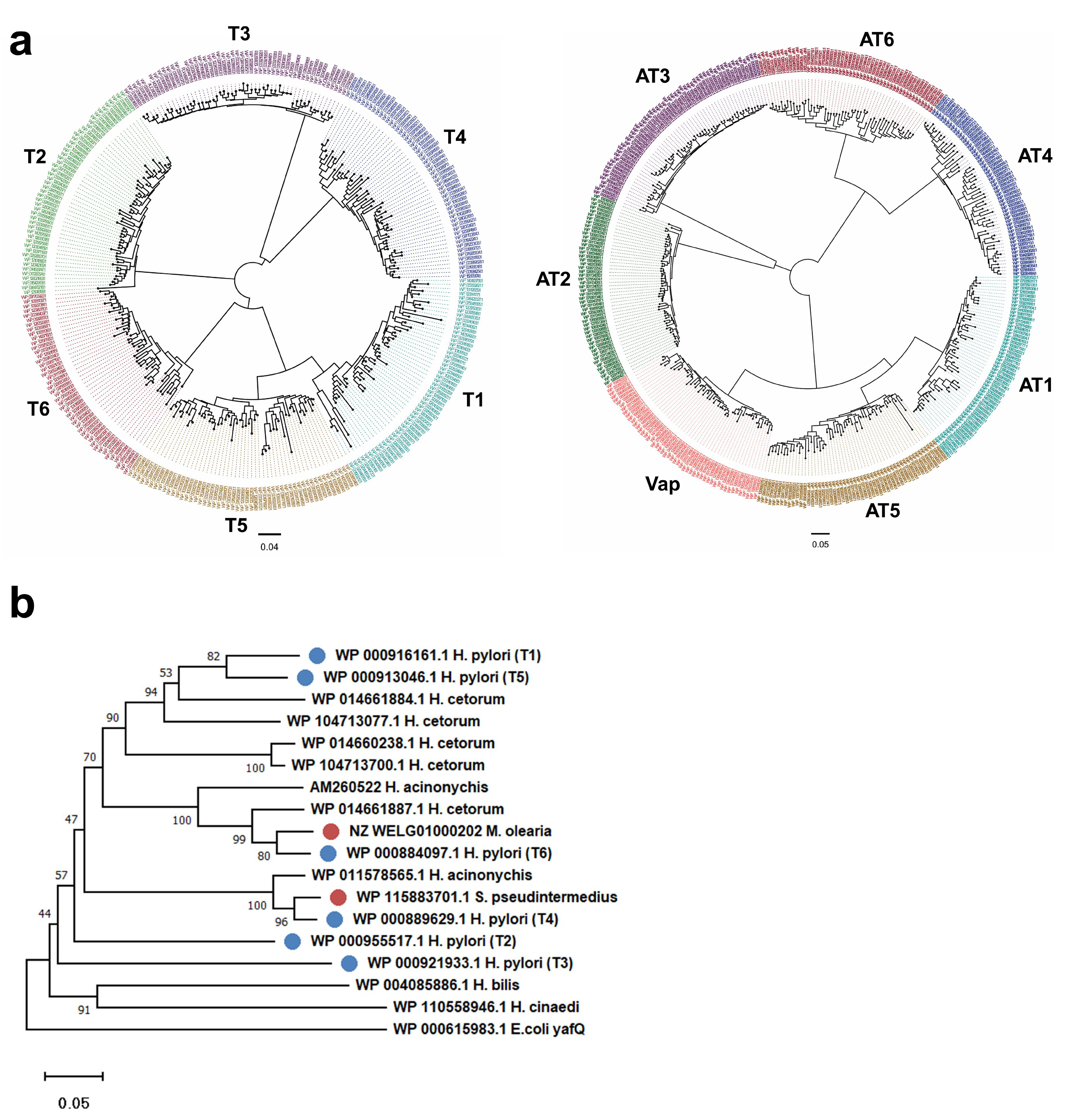


**Supplementary Figure S1.** Identification of *H. pylori* YafQ-family clades and closely related sequences. **(a)** Phylogeny of TA module sequences from diverse *H. pylori* strains. Fifty sequences representative of different toxin (T1-T6) or antitoxin (AT1-AT6) clades were selected from a curated collection of 707 BLASTp consensus sequence hits for analysis using the Neighbour-Joining method. **(b)** Phylogeny of toxin sequences from defined *H. pylori* clades (highlighted with blue circles) and closely related species, *H. acinonychis* and *H. cetorum*. Outgroup clustering of YafQ-family toxins from all other *Helicobacter* species is represented by *H. bilus* and *H. cinaedi*. Highly similar toxin sequences identified in the genome of the Bacteroidetes bacterium *Muricauda olearia* and a plasmid of the Firmicute *Staphylococcus pseudintermedius* strains ST64, ST283, ST496, and ST525 are highlighted with red circles. The percentage of replicate trees in which the associated sequences clustered together in the bootstrap test (1000 replicates) are shown next to the branches.


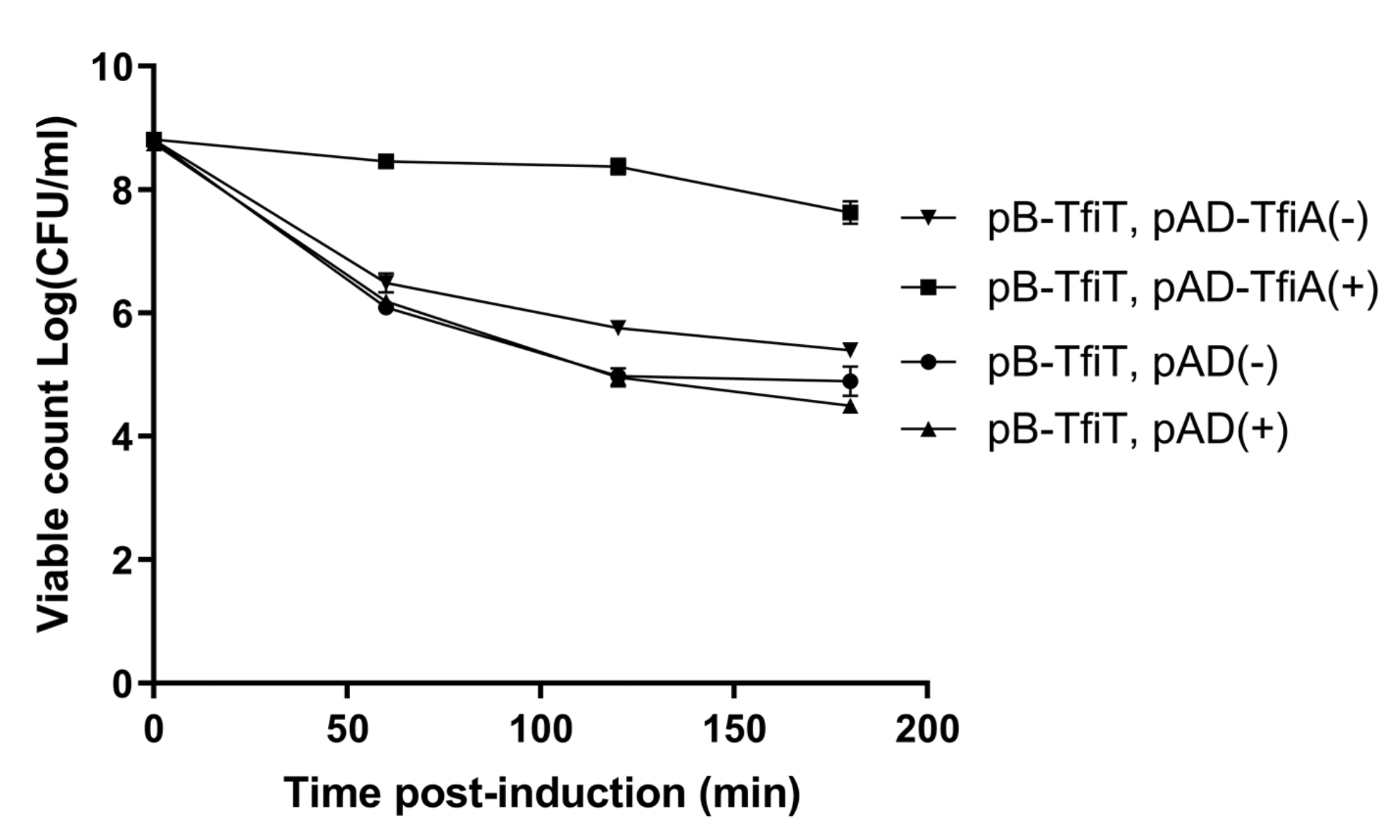


**Supplementary Figure S2.** Delayed expression of TfiA rescues TfiT-mediated growth arrest. Sample dilutions of cultures containing co-resident pBAD-TfiT (arabinose induced) and IPTG-inducible pACYCDuet-TfiA or pACYCDuet control plasmids were plated on LB-ampicilin plates with (+) or without (-) IPTG and incubated at 37^o^C overnight for determination of colony forming units. Error bars display standard deviations from three replicates. pB; pBAD18, pAD; pACYCDuet-1.


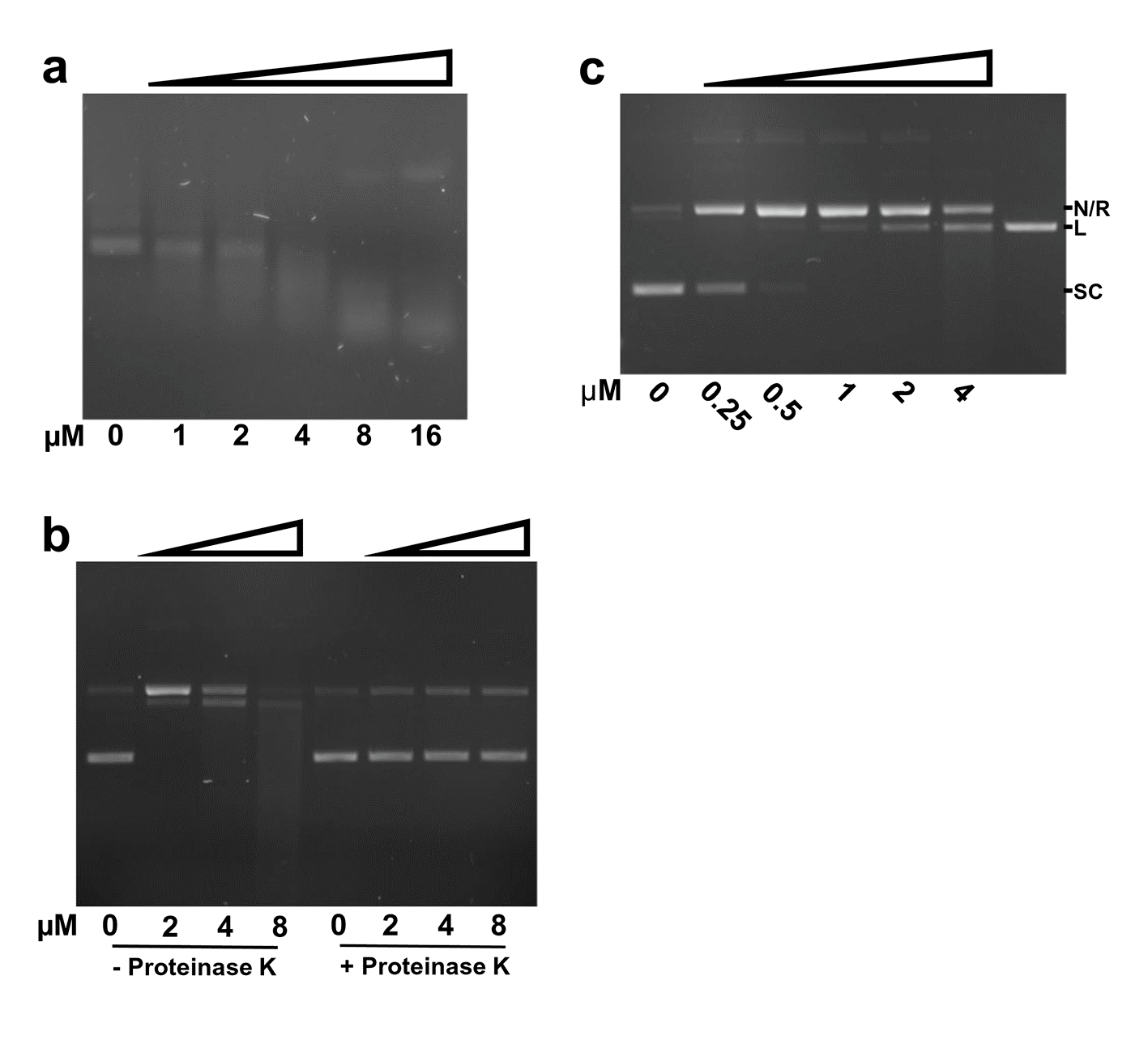


**Supplementary Figure S3.** Sensitivity and specificity of TfiT endonuclease activity**. (a)** Incubation of purified TfiT-His (1-16 µM) with *tfiT* mRNA (100 ng) **(b)** Incubation of Proteinase K treated or untreated TfiT-His (0.25 – 4 µM) with pGEM plasmid DNA (100 ng). **(c)** Incubation of purified TfiT-His (0.25 – 4 µM) with pGEM plasmid DNA (100 ng). A sample of NcoI-restricted linear plasmid DNA (30 ng) is included as a size marker in the final lane. Labelled bands in **(c)** correspond to nicked/relaxed (N/R), linear (L) and supercoiled (SC) plasmid forms.

**
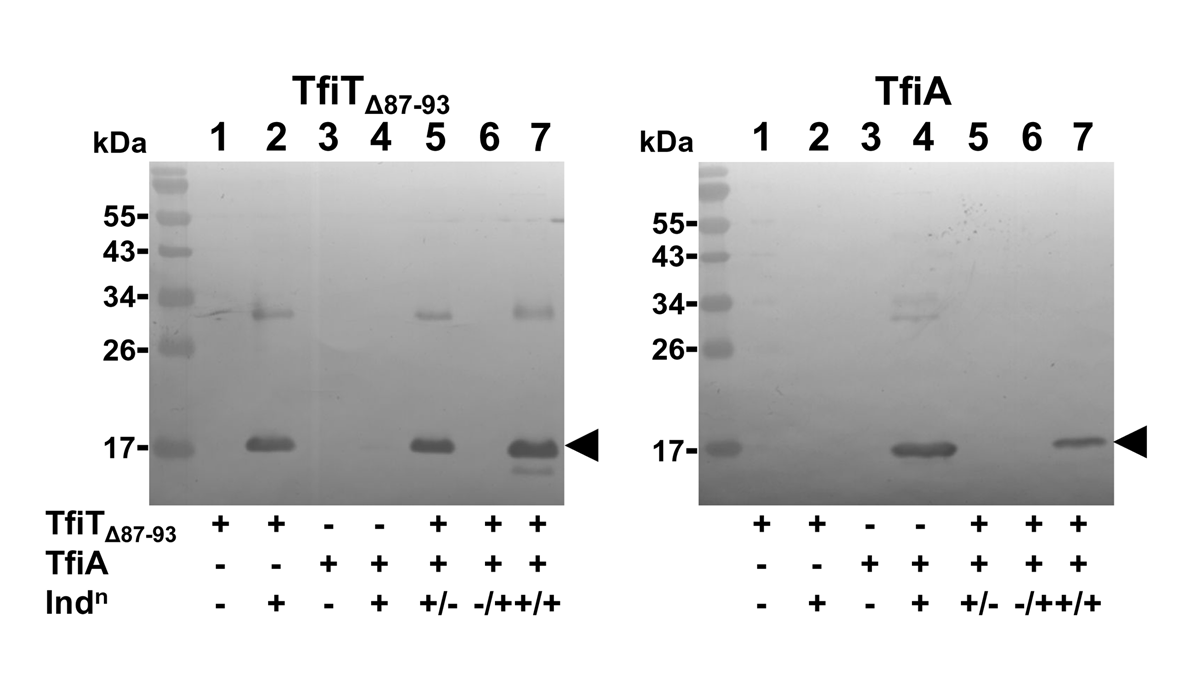
**

**Supplementary Figure S4.** TfiT C-terminal deletion does not abrogate interaction with TfiA.

In parallel column-pulldowns, clarified lysate from induced (lanes 5, 7) or uninduced (lane 6) cultures of pBAD-TfiT_Δ87-93_ were passed over TALON® resin prior to addition of lysate from a culture of either induced (lanes 6, 7) or uninduced (lane 5) S-tagged-TfiA. Elution fractions were assessed for co-purified protein in Western immunblots probed with either anti-His-tag (left panel) or anti-S-tag antibody (right panel). Samples from uninduced/induced cultures for both TfiT (lanes 1, 2 respectively) and TfiA (lanes 3, 4 respectively) were included to monitor expression. Arrowheads indicate TA proteins. Ind^n^ – induction.


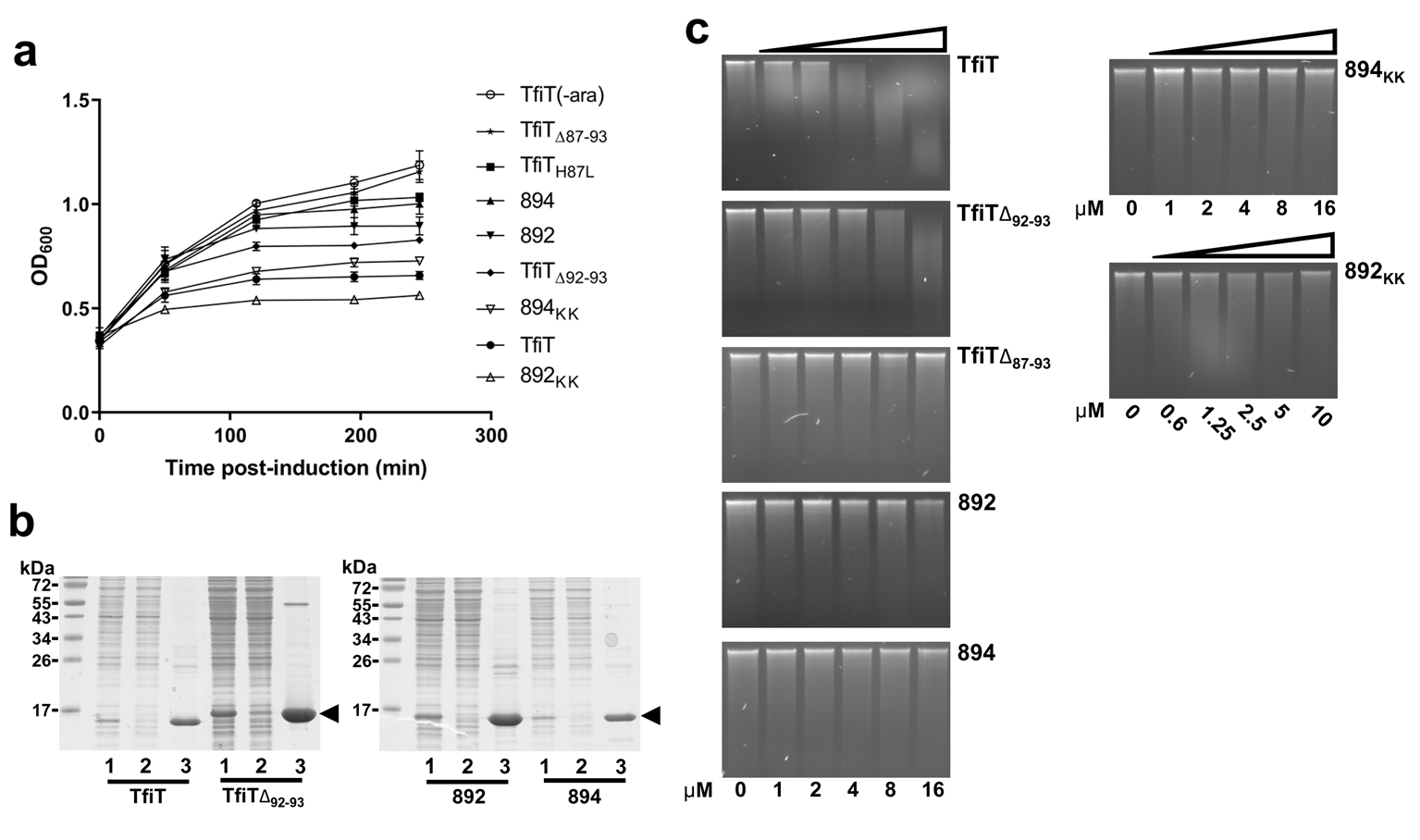


**Supplementary Figure S5.** Growth inhibitory and endonuclease activity of Clade 1, 2 and 3 toxins and mutants. **(a)** Growth profiles of TfiT, HP0892 and HP0894 and their respective mutants up to 3 hrs following induction of toxin expression. **(b)** Representative Coomassie-stained SDS-PAGE gels showing bacterial lysates containing the indicated His-tagged toxins before (lanes 1) and after (lanes 2) incubation with Talon resin, and following elution (lanes 3). Arrowheads indicate TA proteins. **(c)** Incubation of purified His-tagged toxins and mutants (1 - 16 µM) with genomic DNA of *H. pylori* strain P12 (100 ng) in reaction buffer (20 mM Tris-HCl pH 7.0, 50 mM NaCl, 2.5 mM MgSO_4_) at 37^o^C for 30 min. Error bars in **(a)** display standard deviations from three replicates.


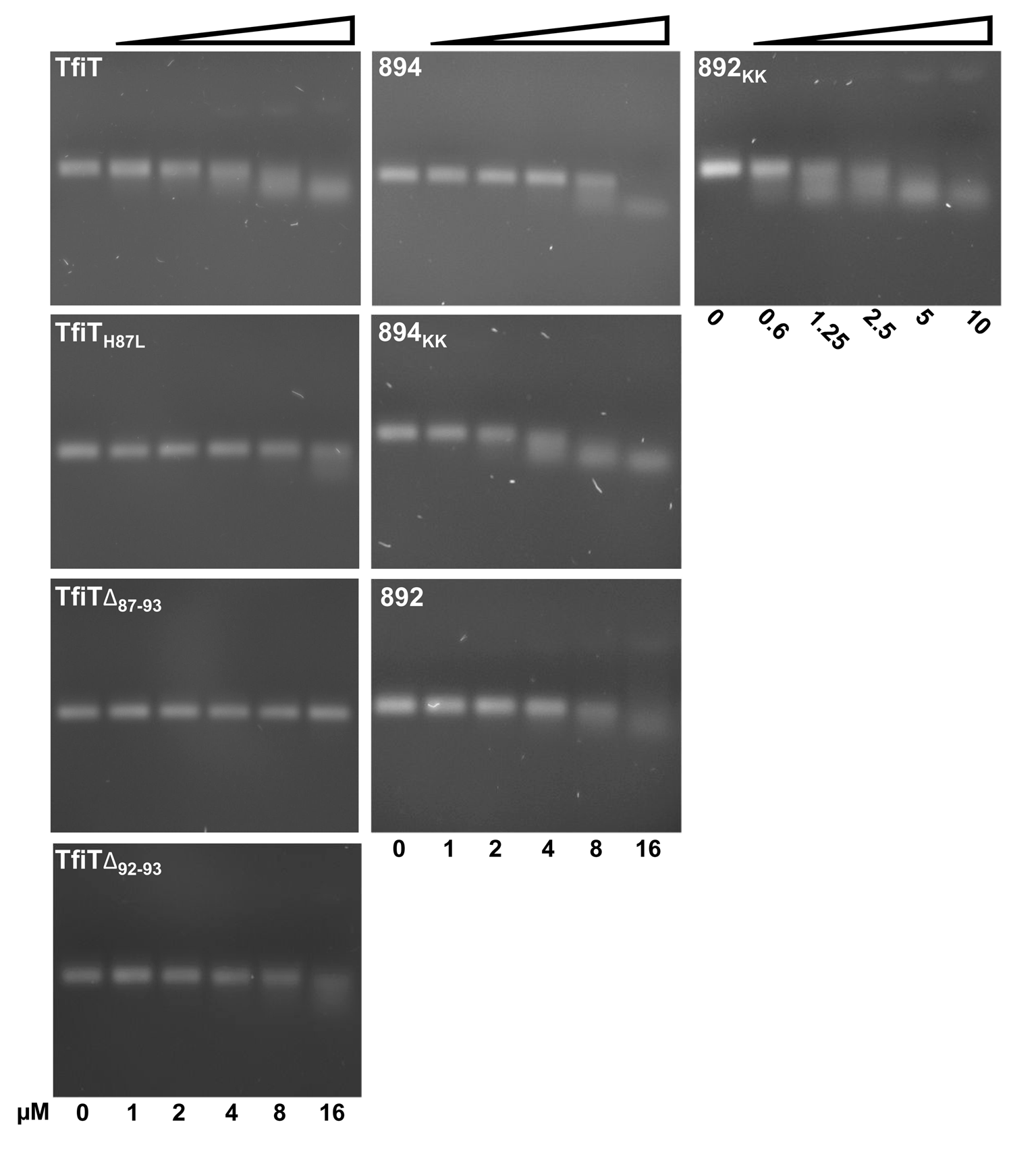


**Supplementary Figure S6.** Full-length gels shown in respective panels of Figure 5b and 5d showing incubation of purified TfiT-His and C-terminal mutant derivatives (1 - 16 µM) or TfiT-His, HP0892-His, HP0894-His and their respective C-terminal lysine-augmented or depleted derivatives (1 - 16 µM) with *H. pylori recA* mRNA (100 ng).

**
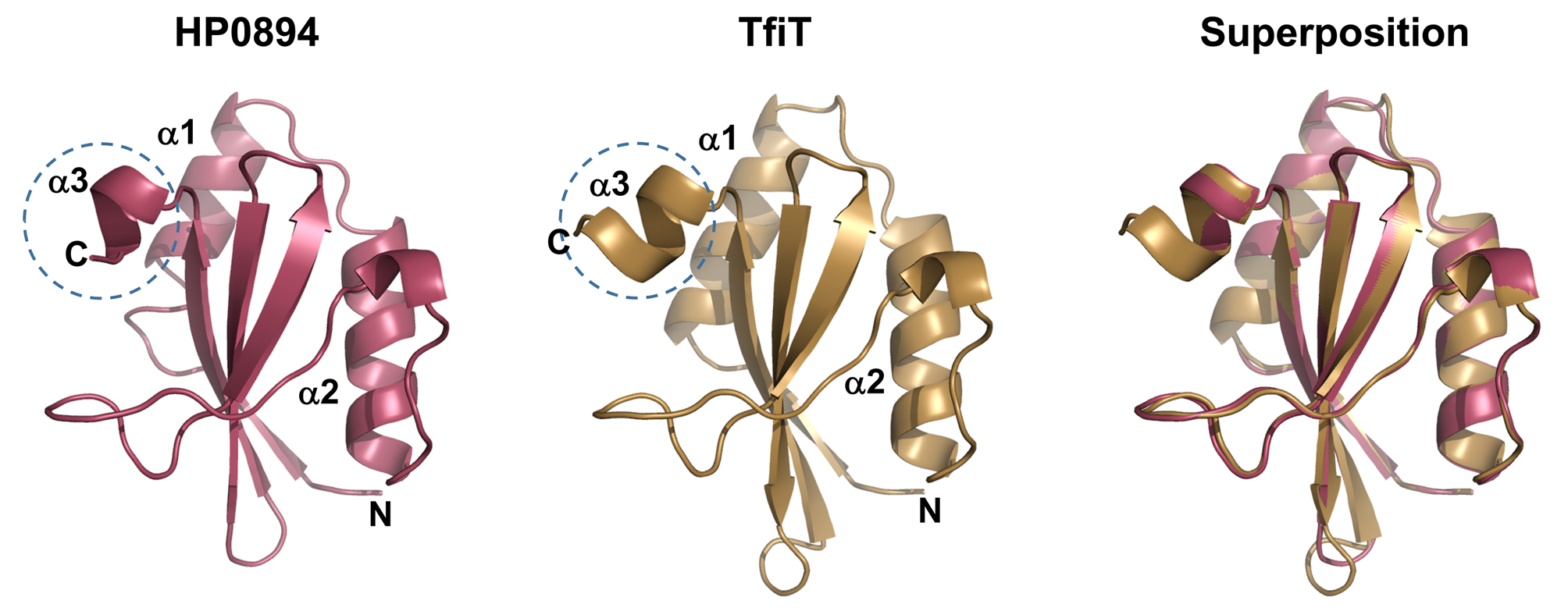
**

**Supplementary Figure S7.** Structural homology of TfiT with genomic toxins HP0892 and HP0894. Ribbon representation of the crystal structure of *H. pylori* HP0894 (PDB: 4LTT) used as template for generation of the TfiT structural homology model.

| **Strains** | **Relevant genotype and/or description** | **Reference or source** |
| --- | --- | --- |
| **Strains** |  |  |
| *S. cerevisiae* PJ69-4A | (*MAT*α *trp1–901 leu2–3112 ura3–52 his3–200 GAL4*Δ *gal80*Δ *LYS2::GAL1-HIS3 GAL2-ADE2* *met2::GAL7-lacZ*) | 67 |
| *E. coli* XLI-Blue | *recA1 endA1 gyrA96 thi-1 hsdR17 supE44 relA1 lac* [F´ *proAB lacI*q*ZΔM15* Tn*10* (Tetr)]. | Agilent Technologies |
| *E. coli* MG1655 | K-12 F– *λ– ilvG– rfb-50 rph-1* | 68 |
| *E. coli* BW25113 | *lacI+rrnBT14 ΔlacZWJ16 hsdR514 ΔaraBADAH33 ΔrhaBADLD78 rph-1, Δ(araB–D)567 Δ(rhaD–B)568 ΔlacZ4787(::rrnB-3) hsdR514 rph-1* | 69 |
| *E. coli* BL21(DE3) pLysS | *F– ompT gal dcm lon hsdSB(rB–mB–) λ(DE3 [lacI lacUV5-T7p07 ind1 sam7 nin5]) [malB+]K-12(λS) pLysS[T7p20 orip15A](CmR)* | Promega |
| *E. coli* SoluBL21 | F- *ompT hsd*S_B_ (r_B_- m_B_- ) *gal dcm* (DE3) | Amsbio |
| *H. pylori* P12 | Reference strain | 70 |
| *H. pylori* HP26695 | Reference strain | 71 |

**Supplementary Table S1.** Strain genotypes

| **Plasmids/constructs** | **Description** | **Reference or source** |
| --- | --- | --- |
| pGEM-T Easy | High copy number cloning vector, Ap^R^ | Promega |
| pBAD/*myc*-His | *ara*BAD promotor for tightly regulated expression, Ap^R^ | Invitrogen |
| pACYCDuet-1 | Dual multiple cloning site co-expression vector, Cm^R^ | Novagen |
| pETDuet-1 | Dual multiple cloning site co-expression vector, Ap^R^ | Novagen |
| pGAD424 | *ori*ColE1 *ori*2µ LEU1 P_ADH_::GAL4 activator domain::MCS | 67 |
| pGBT9 | *ori*ColE1 *ori*2µ TRP1 P_ADH_::GAL4 binding domain::MCS | 67 |
| pUC18 | High copy number cloning vector, Ap^R^ | Pharmacia |
| pBAD-*tfiT* | Overexpression of C-terminal His-tagged TfiT | This study |
| pBAD-*tfiA* | Overexpression of C-terminal His-tagged TfiA | This study |
| pACYCDUET-*tfiA* | Overexpression of C-terminal S-tagged TfiA | This study |
| pACYCDUET-TA module | Construct for overexpression of N-terminal His-tagged TfiA and C-terminal S-tagged TfiT | This study |
| pBAD-*tfiT*_H87L_ | Overexpression of C-terminal His-tagged TfiT_H87L_ | This study |
| pBAD-*tfiT*_Δ87-93_ | Overexpression of C-terminal His-tagged TfiT _Δ87-93_ | This study |
| pBAD-*tfiT* _Δ92-93_ | Overexpression of C-terminal His-tagged TfiT _Δ82-93_ | This study |
| pBAD-*hp0892* | Overexpression of C-terminal His-tagged HP0892 | This study |
| pBAD-*hp0894* | Overexpression of C-terminal His-tagged HP0894 | This study |
| pBAD-*hp0892*_KK_ | Overexpression of C-terminal His-tagged HP0892_KK_ | This study |
| pBAD-*hp0894*_KK_ | Overexpression of C-terminal His-tagged HP0894_KK_ | This study |
| pGAD424-*tfiA* | Fusion of *tfiA* (1 – 315bp) with GAL4 activation domain | This study |
| pGAD424-*tfiT* | Fusion of *tfiT* (1 – 282bp) with GAL4 activation domain | This study |
| pGBT9-*tfiA* | Fusion of *tfiA* (1 – 315bp) with GAL4 binding domain | This study |
| pGBT9-*tfiT* | Fusion of *tfiT* (1 – 282bp) with GAL4 binding domain | This study |
| pUC18-pF0-TA | *tfs4* TA module without upstream flank | This study |
| pUC18-pF1-TA | *tfs4* TA module including 213bp upstream flank | This study |
| pUC18-pF2-TA | *tfs4* TA module including 160bp upstream flank | This study |

**Supplementary Table S2.** Plasmids and constructs

| **Name** | **Oligonucleotide sequence** |
| --- | --- |
| F: pBAD-*tfiT* | GAACTGCCATGGGTCTTAAAGTCAGAACTAAGAAAGATTTTC |
| R: pBAD-*tfiT* | GTACTTGAATTCCTTTTTAAATAGCTCGCTATGAGTG |
| F: pBAD-*tfiA* | GAACTGCCATGGGTTCAAATGTTATTACAGATTACTCGC |
| R: pBAD-*tfiA* | GTACTTGAATTCAGCATTATGCCTTTCTCTTTCTTG |
| R: pBAD-*tfiT*_H87L_ | GTACTTGAATTCCTTTTTAAATAGCTCGCTAAGAGTG |
| R: pBAD-*tfiT*_Δ87-93_ | GTACTTGAATTCAGTGTCTAGACGCAATAAAACAAG |
| R: pBAD-*tfiT* _Δ92-93_ | GTACTTGAATTCAAATAGCTCGCTATGAGTGTCTAG |
| F: pBAD-*hp0892* | GAACTGCCATGGGGCTGACGATTGAAACCAGTAA |
| R: pBAD-*hp0892* | GTACTTGAATTCAAACAGCTCGCTATGACTGC |
| F: pBAD-*hp0894* | GAACTGCCATGGGGTTGAAGCTCAATCTTAAAAAATCT |
| F: pBAD-*hp0892*_KK_ | GTACTTGAATTCCTTTTTAAACAGCTCGCTATGACTGC |
| F: pACYCDUET-*tfiA* | GAACTGCATATGTCAAATGTTATTACAGATTACTCG |
| R: pACYCDUET-*tfiA* | GAACTGCTCGAGAGCATTATGCCTTTCTCTTTCTTG |
| F: pACYCDUET-*tfiA/tfiT* | GAACTTGGATCCGTCAAATGTTATTACAGATTACTCGC |
| R: pACYCDUET-*tfiA/tfiT* | GAACTGCTCGAGCTTTTTAAATAGCTCGCTATGAGTG |
| F: pGAD424- *tfiA* | TTACCGGAATTCTCAAATGTTATTACAGATTACTCGC |
| R: pGAD424- *tfiA* | GAACTTGTCGACTTAAGCATTATGCCTTTCTCTTTC |
| F: pGAD424- *tfiT* | TTACCGGAATTCCTTAAAGTCAGAACTAAGAAAGATTTTC |
| R: pGAD424- *tfiT* | GAACTTGTCGACTCACTTTTTAAATAGCTCGCTATG |
| F: pUC18-pF0 | GAACTGGAGCTCATGTCAAATGTTATTACAGATTACTCG |
| F: pUC18-pF1 | GAACTGGAGCTCAAAGTGCAGGATAACACTTCAA |
| F: pUC18-pF2 | GAACTGGAGCTCCAGGTGGCTAAAACACGCA |
| F: T7-*recA* | GAACTGCCATGGTAATACGACTCACTATAGGGATGGCAATAGATGAAGACAAACA |
| R: *recA* | CGGGTCGACTTATTCCATTTCTTCTAAAGGCTC |
| F: T7-*tfiT* | GAACTGCCATGGTAATACGACTCACTATAGGGATGCTTAAAGTCAGAACTAAGAAAG |

**Supplementary Table S3.** Oligonucleotides

| Bait^a^ | TfiA | | TfiT_Δ87-93_ |
| --- | --- | --- | --- |
| Prey^b^ | TfiT_H87L_ | TfiT_Δ87-93_ | TfiA |
| -His/-Ade^c^ | +++ | +++ | +++ |
| -His^c^ | +++ | +++ | +++ |
| *lacZ*^d^ (Miller Units) | 66.4±8.5 | 87.9±4.7 | 63.3±4.4 |
| *lacZ*^d^ (fold increase)^e^ | 15.6 | 20.7 | 30.1 |

^a^Bait and ^b^prey fusions were constructed in pGBT9 and pGAD424 vectors respectively. ^c^YMM plates were supplemented with Met and uracil and lacked either His, or both His and Ade as indicated. ^d^*lacZ* reporter activity was assessed by β-galactosidase assay. ^e^fold increase relative to self-activation control for each bait vector.

**Supplementary Table S4.** Interaction of TfiT C-terminal mutant with TfiA by yeast-two hybrid assay

**Supplementary References**

67 James, P., Halladay, J. & Craig, E. A. Genomic libraries and a host strain designed for highly efficient two-hybrid selection in yeast. Genetics 144, 1425-1436 (1996).

68 Hayashi, K. et al. Highly accurate genome sequences of Escherichia coli K-12 strains MG1655 and W3110. Mol Syst Biol 2, 2006 0007, doi:10.1038/msb4100049 (2006).

69 Datsenko, K. A. & Wanner, B. L. One-step inactivation of chromosomal genes in Escherichia coli K-12 using PCR products. Proc Natl Acad Sci U S A 97, 6640-6645, doi:10.1073/pnas.120163297 (2000).

70 Fischer, W. et al. Strain-specific genes of Helicobacter pylori: genome evolution driven by a novel type IV secretion system and genomic island transfer. Nucleic Acids Res 38, 6089-6101, doi:10.1093/nar/gkq378 (2010).

71 Tomb, J. F. et al. The complete genome sequence of the gastric pathogen Helicobacter pylori. Nature 388, 539-547, doi:10.1038/41483 (1997).
